# Strategies for Sustainable Production of Hydrogen Peroxide via Oxygen Reduction Reaction: From Catalyst Design to Device Setup

**DOI:** 10.1007/s40820-023-01067-9

**Published:** 2023-05-09

**Authors:** Yuhui Tian, Daijie Deng, Li Xu, Meng Li, Hao Chen, Zhenzhen Wu, Shanqing Zhang

**Affiliations:** 1 School of Chemical Engineering and Light Industry, Guangdong University of Technology, Guangzhou, 510006 People’s Republic of China; 2 Centre for Catalysis and Clean Energy, School of Environment and Science, Griffith University, Gold Coast Campus, Gold Coast, Queensland 4222 Australia; 3 Institute for Energy Research, School of Chemistry and Chemical Engineering, Key Laboratory of Zhenjiang, Jiangsu University, Zhenjiang, 212013 People’s Republic of China

**Keywords:** Hydrogen peroxide, Electrochemical synthesis, Electrocatalysts, Sustainable technologies

## Abstract

An environmentally benign, sustainable, and cost-effective supply of H_2_O_2_ as a rapidly expanding consumption raw material is highly desired for chemical industries, medical treatment, and household disinfection. The electrocatalytic production route via electrochemical oxygen reduction reaction (ORR) offers a sustainable avenue for the on-site production of H_2_O_2_ from O_2_ and H_2_O. The most crucial and innovative part of such technology lies in the availability of suitable electrocatalysts that promote two-electron (2e^–^) ORR. In recent years, tremendous progress has been achieved in designing efficient, robust, and cost-effective catalyst materials, including noble metals and their alloys, metal-free carbon-based materials, single-atom catalysts, and molecular catalysts. Meanwhile, innovative cell designs have significantly advanced electrochemical applications at the industrial level. This review summarizes fundamental basics and recent advances in H_2_O_2_ production via 2e^–^-ORR, including catalyst design, mechanistic explorations, theoretical computations, experimental evaluations, and electrochemical cell designs. Perspectives on addressing remaining challenges are also presented with an emphasis on the large-scale synthesis of H_2_O_2_ via the electrochemical route.

## Highlights

- The state-of-the-art development in electrochemical H_2_O_2_ production via the two-electron oxygen reduction reaction is reviewed with emphasis on material science, reaction mechanisms, and fundamental factors that govern the reaction route.
- General principles and strategies for catalyst design are summarized to understand the inherent relationships between the catalyst properties and electrocatalytic performances.
- Perspectives and challenges are presented to get insights into the large-scale manufacturing of H_2_O_2_ via the electrochemical routes.

## Graphical abstract

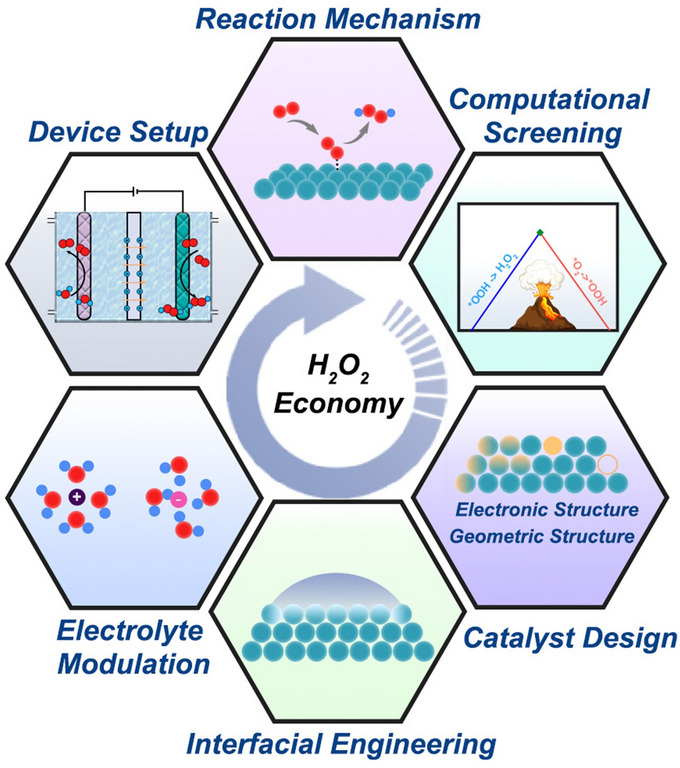

## Introduction

**Fig. 1:**
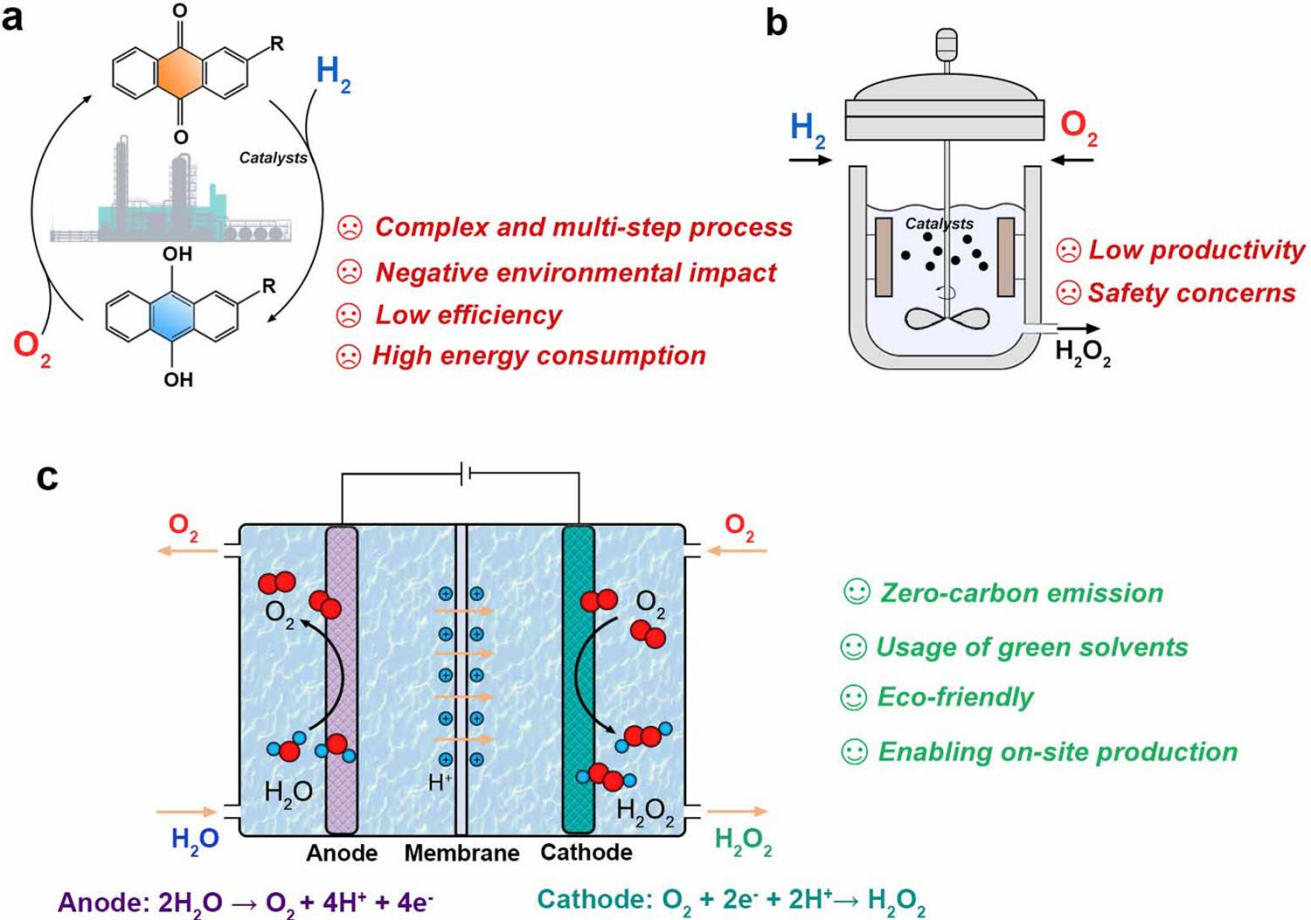
Schematic diagrams of H_2_O_2_ production strategies via **a** classic anthraquinone process, **b** direct synthesis from H_2_ and O_2_, and **c** electrocatalytic synthesis via 2e^–^-ORR

Hydrogen peroxide (H_2_O_2_) is an important chemical in environmental and energy applications. It is an eco-friendly oxidant widely used in the household, medical, agricultural, and industries, such as environmental purification, chemical synthesis, and textile bleaching [1]. For example, the World Health Organization (WHO) has listed H_2_O_2_ as the crucial disinfectant against the current COVID-19 pandemic [2]. Moreover, H_2_O_2_ is a sustainable energy carrier. When used as a fuel, the only products are water, oxygen, and heat with zero carbon emissions [3, 4]. Since H_2_O_2_ can serve as both fuel and oxidant in the fuel cell system, it is a powerful fuel in aerospace for rocket propulsion [5, 6]. With the rapidly growing demand, it is predicted that the overall market demand will increase to 5.7 million metric tons by 2027 [7]. However, industrial H_2_O_2_ manufacture is almost exclusively through the anthraquinone method, involving the sequential hydrogenation and oxidation of anthraquinone molecules (Fig. 1a) [1, 8]. The H_2_O_2_ is extracted from the organic working solution, leaving the solvent/anthraquinone mixture to be recycled. This process is able to produce an average yield of 50 thousand tons per year per plant and accounts for the largest part of the world’s H_2_O_2_ production [9].

Unfortunately, the conventional anthraquinone method has several drawbacks: (1) The multiple steps and complexity of this process require costly and large-scale equipment/infrastructure. (2) The formation of unwanted organic byproducts and substantial wastes will negatively impact the environment. (3) The limited solubility of anthraquinone, uncontrollable side reactions, and mass-transport limitation between hydrogenation and oxidation reactors together reduce productivity and efficiency. (4) The subsequent separation, concentration, and purification of the produced pure H_2_O_2_ are time-consuming and associated with extra energy consumption. Thus, such an energy-intensive process cannot meet the standard of sustainable and low-carbon chemical production.

The straightforward method for synthesizing H_2_O_2_ is direct from H_2_ and O_2_ (Fig. 1b) [1]. The reaction, H_2_ + O_2_ → H_2_O_2_ with a standard Gibbs free formation energy, Δ*G*_0_^f^ =  −120 kJ mol^−1^, is exergonic and thermodynamically favored [9]. The high atom economy and nontoxic byproduct (only water) make it non-pollution to the environment. However, the unwanted oxidation of H_2_ to H_2_O instead of H_2_O_2_ severely reduces the production yield [10]. Although extensive research has been demonstrated on Pd-based catalysts to enhance catalytic selectivity, safety concerns should not be neglected due to the explosive nature of the H_2_/O_2_ mixture, which hinders the practical feasibility of this method [8]. These concerns motivate alternative synthetic technologies with minimized environmental impact, improved safety, reduced energy demand, and process cost.

In recent years, the development of advanced electrode materials and electrochemical cell devices enables the production of valuable chemicals (e.g., H_2_, NH_3_, Cl_2_, and C_*x*_H_*y*_O_*z*_) from cheap and earth-abundant reactants via the electrochemical way [11]. Particularly, the synthesis of H_2_O_2_ through electrochemical oxygen reduction has proved feasible and achievable [12, 13]. In a typical membrane-based flow cell reactor (Fig. 1c), the cathode and anode are separated by the membrane, allowing proton transport to the cathode. H_2_O_2_ can be produced via the two-electron (2e^–^) reduction of O_2_ at the cathode, while the oxidation reactions, such as the oxygen evolution reaction (OER), occur at the anode. This electrochemical approach has non-organic wastes and a zero-carbon footprint by using O_2_ and H_2_O as raw materials and renewable electricity as the energy input. The operation can be conducted under ambient temperature and pressure. The explosive environment from the H_2_/O_2_ mixture is avoided due to the separation of the anode and cathode. Moreover, the on-site production of dilute H_2_O_2_ can be realized by portable and distributed devices, thus preventing the transportation and storage of hazardous bulk H_2_O_2_ [14]. The production rate can be controlled by adjusting the applied potential and gas/fluid flow [15].

Notably, H_2_O_2_ can also be produced at the anode via the 2e^**–**^-water oxidation reaction (WOR) [16]. The experimental setup is rather simple due to no gas-phase reactant [16]. However, the 2e^**–**^-WOR requires an ultra-high overpotential (above 1.76 V at standard conditions) [17]. The intrinsic instability of H_2_O_2_ during the WOR substantially restricts this route [18, 19]. Thus, the current electro-synthesis of H_2_O_2_ is primarily via the 2e^**–**^-oxygen reduction reaction (ORR). The challenge is that the ORR would go through the competitive four-electron (4e^–^) pathway and produce H_2_O in acid solutions or OH^−^ in alkaline media, causing a selectivity issue [20]. Efficient and robust electrocatalysts with high activity, selectivity, and stability are prerequisites for developing electrochemical H_2_O_2_ production. Specifically, the high activity means that ORR can proceed with a low overpotential and fast kinetics, while the high selectivity enables the high production yield of H_2_O_2_ via the 2e^–^-process. High stability can ensure sustainable and long-lasting H_2_O_2_ production without degradation [21].

Over past decades, substantial efforts have been devoted to 4e^–^-ORR electrocatalysts for applications in fuel cells and metal-air batteries due to their high efficiency for generating electricity, while the 2e^–^-pathway has been regarded as a side-reaction to be avoided [22]. As electrochemical H_2_O_2_ production has received surging interest, various 2e^–^-ORR catalysts have been purposely designed and systemically investigated. To further advance this burgeoning field, it is necessary to understand the intrinsic relationship between the enhanced catalytic performance and the as-designed catalyst.

This review covers the state-of-the-art development of 2e^–^-ORR electrocatalysts that are selective for electrochemical H_2_O_2_ production and emphasizes material science, reaction mechanisms, and fundamental factors that govern the reaction route. It begins with the fundamental basics of electrochemical ORR, including possible reaction mechanisms, experimental methods for evaluating catalysts’ properties, and H_2_O_2_-quantification methods. Theoretical understanding of the ORR process on the atomic level will be discussed. The currently applied concepts and general principles for material design will be summarized to understand the correlation between the properties of catalyst surfaces and electrocatalytic performances. Subsequently, different categories of catalyst materials and corresponding design strategies will be reviewed to illustrate how these principles and strategies guide the rational design of optimal 2e^–^-ORR electrocatalysts. Importantly, influences of interfacial factors and reaction medium (e.g., pH, the composition of cations and anions) on the H_2_O_2_ production will also be included to get insights into optimizing the catalysis system for high-rate conversion. Beyond the fundamental research, the potential applications integrated with electrochemical H_2_O_2_ generation devices will be introduced. The remaining challenges and perspectives on future directions are provided at the end to guide future development in the electrochemical synthesis of H_2_O_2_.

## Fundamentals for ORR

The electrochemical ORR is a complex process involving multistep electron transfer and various reactive intermediates [23]. A fundamental understanding of the catalytic behaviors and reaction mechanisms on the catalyst surface is essential for the rational design of suitable electrocatalysts for targeted reaction routes.

### ORR Pathways: 2e^–^ versus 4e^–^

During the electrochemical ORR, the O_2_ molecule is first adsorbed on the catalyst surface, which can be completely reduced to H_2_O/OH^−^ via the 4e^–^-process (dissociation of O–O bond) or be incompletely reduced to H_2_O_2_/HO_2_^−^ through a 2e^–^-pathway [24]. The overall reactions are presented as follows [17]:

In acid electrolytes:

1 $${\text{O}}_{2} + 4{\text{H}}^{ + } + 4{\text{e}}^{ - } \to 2{\text{H}}_{2} {\text{O}}$$

2 $${\text{O}}_{2} + 2{\text{H}}^{ + } + 2{\text{e}}^{ - } \to {\text{H}}_{2} {\text{O}}_{2}$$

In alkaline electrolytes:

3 $${\text{O}}_{2} + 2{\text{H}}_{2} {\text{O}} + 4{\text{e}}^{ - } \to 4{\text{OH}}^{ - }$$

4 $${\text{O}}_{2} + {\text{H}}_{2} {\text{O}} + 2{\text{e}}^{ - } \to {\text{HO}}_{2}^{ - } + {\text{OH}}^{ - }$$

**Fig. 2:**
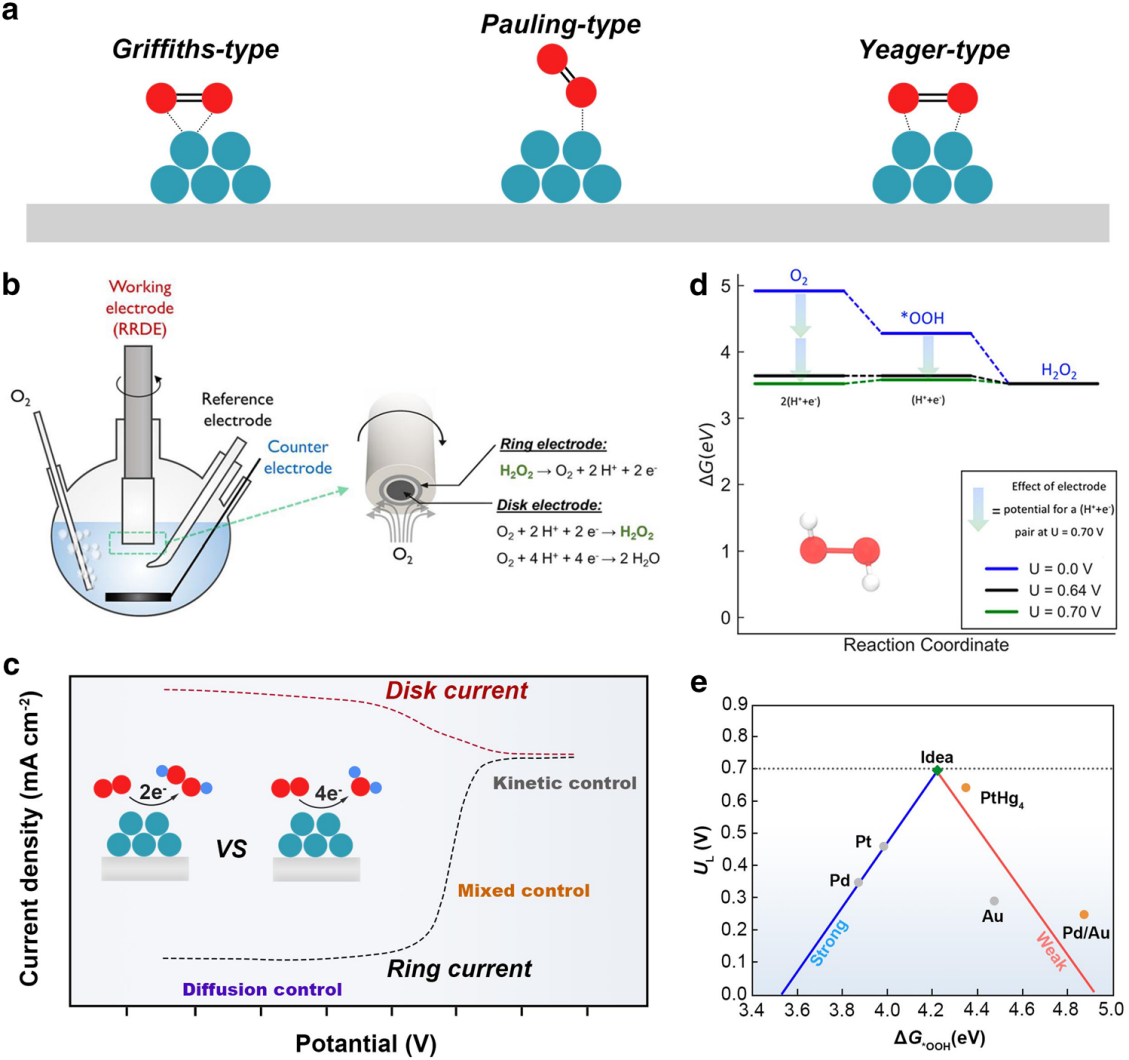
ORR mechanisms and experimental and theoretical evaluations: **a** Possible configurations of O_2_ adsorption on the catalyst surface. **b** Schematic of the RRDE setup in a three-electrode electrochemical cell. Reproduced with permission [28]. Copyright 2018, American Chemical Society. **c** Typical LSV curve obtained on RRDE for ORR. **d** Free energy diagram for the 2e^–^-ORR on PtHg_4_. Reproduced with permission [29]. Copyright 2018, American Chemical Society. **e** A volcano plot of theoretical limiting potential (*U*_L_) as a function of ΔG_*OOH_ for the 2e^–^-ORR. Data adapted from Refs. [7, 9]

In actual electrocatalysis systems, both 2e^–^ and 4e^–^ reactions occur concomitantly and competitively. Since the adsorption of O_2_ is the first step during the ORR process, it mechanistically determines the subsequent reaction route [14]. The adsorption of O_2_ can occur in different ways. As shown in Fig. 2a, three prototypical adsorption configurations have been proposed based on the orientation of adsorbed O_2_ on the catalyst surface [25]. On the bulk catalyst surface, the Griffiths and Yeager models with side-on adsorption of O_2_ might elongate the O–O bond and ease its breaking, thus driving the ORR toward the 4e^–^-pathway [26]. Oppositely, the end-on adsorption mode (“Pauling-type”) of O_2_ is preferred on isolated active sites on the catalyst [27]. The O–O can be kept, thus favoring the selective 2e^–^ reduction with H_2_O_2_ as the final product.

It should be noted that even if H_2_O_2_ is successfully produced via the 2e^–^-pathway, it may undergo further reduction via another 2e^–^-process (Eqs. 5 and 7) and/or a self-decomposition (Eqs. 6 and 8) via the disproportionation reaction. In acid media, it goes as follows:

5 $${\text{H}}_{2} {\text{O}}_{2} + 2{\text{H}}^{ + } + 2{\text{e}}^{ - } \to 2{\text{H}}_{2} {\text{O}}$$

6 $${\text{2H}}_{2} {\text{O}}_{2} \to 2{\text{H}}_{2} {\text{O}} + {\text{O}}_{{2}}$$

In the alkaline solution, it includes the following steps:

7 $${\text{HO}}_{2}^{ - } + {\text{H}}_{2} {\text{O}} + 2{\text{e}}^{ - } \to 3{\text{OH}}^{ - }$$

8 $${\text{2HO}}_{2}^{ - } \to 2{\text{OH}}^{ - } + {\text{O}}_{2}$$

This means that once H_2_O_2_ forms, further reduction or decomposition should also be avoided to enhance productivity [30].

### Experimental Evaluations of 2e^–^-ORR

Similar to that for the 4e^–^-ORR, the laboratory evaluation of the performance of electrochemical H_2_O_2_ production is normally performed in a standard three-electrode system (Fig. 2b). Typically, the rotating ring disk electrode (RRDE) with a platinum ring serves as the working electrode for assessing catalysts’ activity and selectivity. A graphite rod is used as the counter electrode (CE), and a Hg/HgO or Ag/AgCl electrode is implemented as the reference electrode (RE). Catalyst powder is usually dispersed into a homogenous ink with the mixture of water, alcohol (or isopropyl), and Nafion by a certain ratio and then deposited on the glassy carbon disk. The electrocatalytic activity of the working electrode is evaluated by performing linear scan voltammetry (LSV) in O_2_-saturated electrolytes (e.g., 0.1 M HClO_4_ or 0.1 M KOH). A constant potential ranging from 1.2 to 1.3 V (vs. reversible hydrogen electrode (RHE)) is applied to the platinum ring electrode during the test. Oxygen reduction takes place at the disk electrode, generating the cathodic current. H_2_O_2_ produced at the disk electrode is radially transferred to the concentric platinum ring electrode by the forced convection caused by the rotating motion of the electrode. Subsequently, H_2_O_2_ is oxidized back to O_2_ at the ring electrode, giving rise to an anodic current signal as indicative of generated H_2_O_2_.

Figure 2c shows the typical LSV curve recorded on RRDE, which can be divided into three regions corresponding to the electrocatalytic process dominated by different factors [31]. In the beginning, the applied overpotential cannot overcome the reaction barrier. Thus, the reaction rate is relatively slow, and the current density increases slightly as the potential decreases. The limiting factor for the electrochemical reaction is the reaction kinetics. Subsequently, the reaction accelerates with the potential drop due to the far enough potential from the equilibrium potential to drive the electrochemical reaction. When the current remains almost unchanged with increased overpotential, the dissolution and diffusion of oxygen become the limitation, so-called diffusion-controlled zone. Between the kinetic- and diffusion-controlled zone, the current increase rapidly with the potential drop. The ORR is co-dominated by reaction kinetics and mass diffusion, so-called mixed controlled region [31].

The recorded disk (*I*_d_) and ring (*I*_r_) currents are used to quantify the selectivity and average electrons transferred (*n*) during ORR.

The H_2_O_2_ selectivity is calculated using the equation [23]:

9 $$Selectivity \, (\% ) = 200 \times (I_{{\text{r}}} /N)/(I_{{\text{d}}} + I_{{\text{r}}} /N)$$

which represents the fraction of O_2_ consumed at the disk electrode for generating H_2_O_2_.

The corresponding electron transfer number is calculated by the equation:

10 $$n = 4 \times I_{{\text{d}}} /(I_{{\text{d}}} + I_{{\text{r}}} /N)$$

where *N* is the collection efficiency of the platinum ring determined by measuring the redox of hexacyanoferrate ([Fe(CN)_6_]^3−^/[Fe(CN)_6_]^4−^).

To evaluate the amount of H_2_O_2_ generated in synthesis cells or reactors, the catalyst-modified working electrode is typically tested in an electrochemical H-type cell [32]. The working and counter electrodes are separated by Nafion film to avoid further oxidation of H_2_O_2_ on the anode [33]. Over a long-term operation, the stability of the catalyst is examined by measuring current decay, and the produced H_2_O_2_ is accumulated in the electrolyte. The H_2_O_2_ concentration can be roughly estimated by the traditional titration method and colorimetric strips [34]. For more accurate measurement, the conjunction of the titration method and ultraviolet–visible (UV–Vis) spectrophotometry is usually employed. Specifically, cerium sulfate (Ce(SO_4_)_2_) is typically applied as the indicator for H_2_O_2_ in the lab scale. The yellow Ce^4+^ will be reduced by H_2_O_2_ to colorless Ce^3+^.

11 $${\text{H}}_{{2}} {\text{O}}_{{2}} + {\text{2Ce}}^{4 + } \to 2{\text{Ce}}^{3 + } + 2{\text{H}}^{ + } + {\text{O}}_{{2}}$$

The concentration of Ce^4+^ before and after the reaction can be measured by UV–Vis spectroscopy. Hence, the mole of H_2_O_2_ can be determined by measuring the mole of consumed Ce^4+^ [35].

The UV–Vis method is less affected by interference species and exhibits more minor measurement errors, making it an excellent candidate for quantifying accumulated H_2_O_2_ concentrations in an electrochemical environment. In addition, high-performance liquid chromatography (HPLC) enables a rapid and accurate analysis for H_2_O_2_ qualification but may be restricted by the detection limit [4]. Based on the amount of H_2_O_2_ (mg or mmol) generated within a certain period (h^−1^), the mass activity of the electrocatalyst can be presented in the unit of mg or mmol g_cat_^−1^ h^−1^ as an indicator to assess the production rate. The Faradaic efficiency (*λ*%) can be defined as the ratio of charge converted to H_2_O_2_ to the total amount of charge transferred [36]:

12 $$\lambda {\text{ (\% ) = 100}} \times \frac{{n \times F \times {\text{Cumulative H}}_{{2}} {\text{O}}_{{2}} {\text{ yield (mol)}}}}{{\int_{{0}}^{{\text{t}}} {I{\text{ dt}}} }}$$

where *n* is the electron transfer number;* F* is the Faraday constant (96,485.3 C mol^−1^); ${\int }_{0}^{\text{t}}I {\text{d}}{\text{t}}$ is the consumed quantity of electric charge (C).

### Theoretical Computations of 2e^–^-ORR

Computational simulation based on quantum chemistry provides a platform to study the reaction mechanism of a catalytic system and has been applied to guide the search and design of suitable catalyst materials beyond the trial-and-error approach. Through years of investigation, two possible reaction mechanisms for ORR, named association and dissociation mechanisms with successive steps of oxygenated intermediate adsorptions, have been widely accepted as follows [31]:

**Table:** 

Pathways	Acidic solution	Alkaline solution
Associative Pathway [2e^–^]	O_2_ (g) + * + H^+^ + e^−^ ↔ *OOH	O_2_ (g) + * + H_2_O + e^−^ ↔ *OOH + OH^−^
	*OOH + H^+^ + e^–^ ↔ * + H_2_O_2_	*OOH + e^–^ ↔ * + H_2_O
Associative Pathway [4e^–^]	*OOH + H^+^ + e^–^ ↔ *O + H_2_O	*OOH + e^–^ ↔ *O + OH^–^
	*O + H^+^ + e^–^ ↔ *OH	*O + H_2_O + e^–^ ↔ *OH + OH^–^
	*OH + H^+^ + e^–^ ↔ * + H_2_O	*OH + e^–^ ↔ * + OH^–^
Dissociative Pathway [2e^–^ + 2e^–^]	O_2_ (g) + 2* ↔ *O + *O	O_2_ (g) + 2* ↔ *O + *O
	*O + H^+^ + e^–^ ↔ *OH	*O + H_2_O + e^–^ ↔ *OH + OH^–^
	*OH + H^+^ + e^–^ ↔ * + H_2_O	*OH + H_2_O + e^–^ ↔ * + H_2_O + OH^–^

Here, * represents the active site on the catalyst surface. *OOH, *O, and *OH are reaction intermediates during the ORR [31]. Regardless of the associative or dissociative mechanism, *O is one of the dissociation products following the 4e^–^-pathway. Thus, the ability of catalysts to dissociate the O–O bond determines the final reduction products, H_2_O or H_2_O_2_. Catalyst materials with strong oxygen binding energies could be excluded due to favorable *O formation [37, 38]. Accordingly, 2e^–^-ORR involves only one intermediate *OOH, no matter in acid or alkaline media. The adsorption-free energy of *OOH (ΔG_*OOH_) is used to quantify the bond strength and as the key parameter in controlling the 2e^–^-ORR. Thus, adjusting the ΔG_*OOH_ and preventing the dissociation of O–O bond on catalysts to an appropriate level can address the activity and selectivity for the 2e^–^-ORR [39, 40].

Figure 2d shows the typical free energy diagram illustrating the activity of PtHg_4_ catalysts toward H_2_O_2_. At *U* = 0 V, the free energy diagram is downhill, suggesting the energetically favorable process. When the electrode equilibrium potential reaches 0.70 V (the equilibrium potential of the 2e^–^-ORR), the formation of *OOH is slightly uphill, hence the activity-determining factor. The ideal situation is that the Gibbs free energy changes in each elementary step are the same at *U* = 0 V so that all reaction barriers can be zero at the equilibrium potential.

There is a general existence of scaling relations between reactive intermediates, which means that the ORR catalytic pathway can be predicted by simple binding-energy calculation of the key intermediate *OOH. The established activity-volcano plot using ΔG_*OOH_ as the descriptor allows the screening of the optimum catalyst. Figure 2e shows the 2e^–^-ORR volcano plot of the calculated theoretical limiting potential (*U*_L_) on different catalyst surfaces as the function of ΔG_*OOH_. Catalysts at the right leg of the volcano (e.g., Au) exhibit weak *OOH binding and are rate limited by the *OOH formation. Their selectivity for 2e^–^-ORR increases but activity decreases. At the left of the volcano, there are catalysts that bind *OOH strongly (e.g., Pt and Pd). They exhibit a higher driving force to split O–O bond. The apex of the volcano plot, where the most active electrocatalysts are located, is defined as the ideal material with optimal binding of reaction intermediates. Accordingly, the optimal ΔG_*OOH_ value for H_2_O_2_ production is estimated to be 4.22 eV, so the corresponding free energy diagram is flat at 0.70 V [41]. It can be seen that the state-of-the-art 2e^–^-ORR catalyst, PtHg_4_, lies near the top of the volcano, showing good agreement with the experiment [9]. Additionally, the activation energy for the *OOH dissociation can be estimated via the climbing image nudged elastic band (CI-NEB) method [42]. Via computational screening, the catalyst surface with suitable *OOH adsorption and higher energy barrier for dissociation of O–O favors the selective 2e^–^-ORR.

### General Principles and Engineering Strategies

**Fig. 3:**
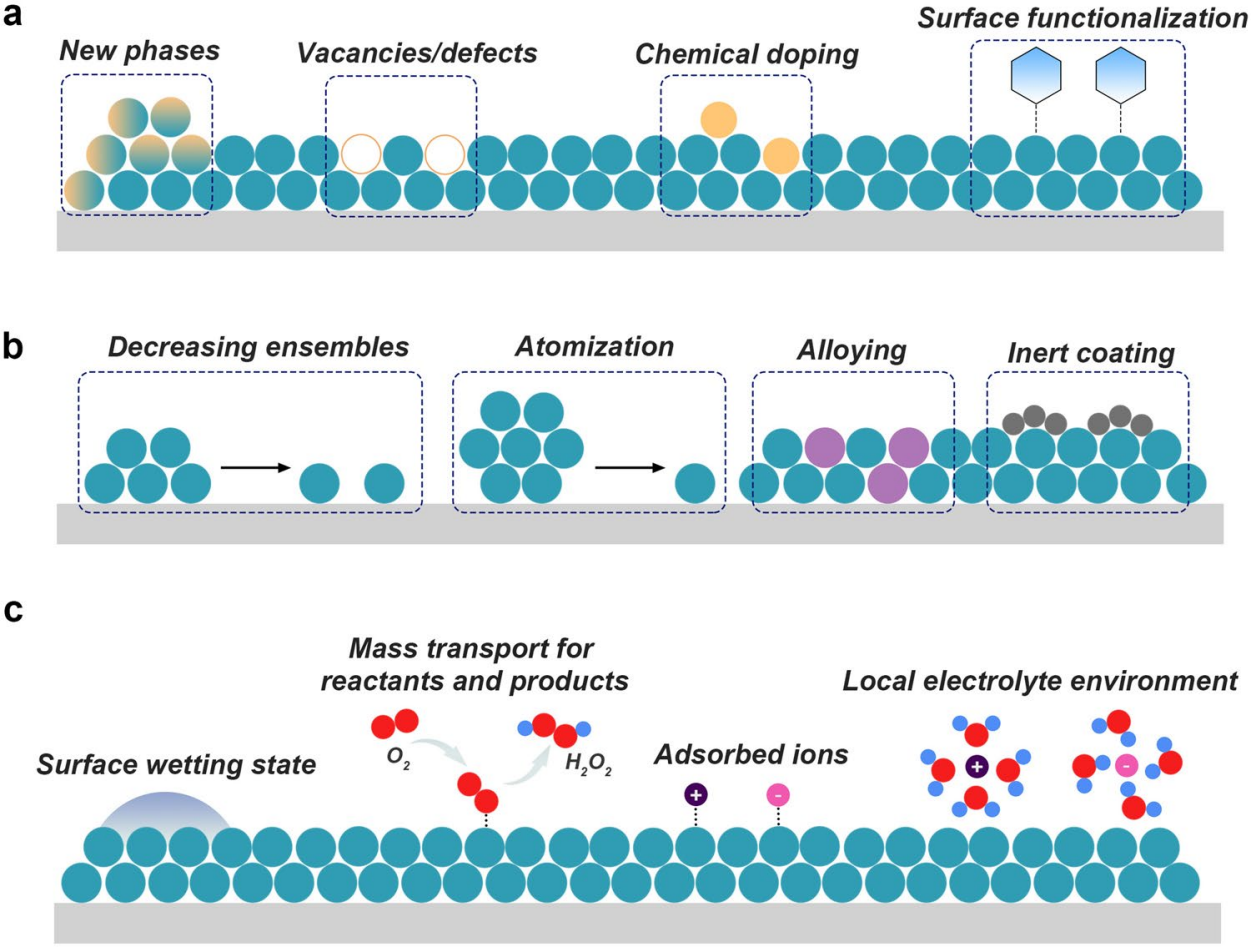
The designs of electrocatalysts for 2e^–^-ORR: **a** Schematic illustration of engineering electrocatalysts via facet engineering, defect engineering, chemical doping, and surface functionalization. **b** Schematic illustration of site isolating via decreasing ensembles, atomization, alloying with a secondary metal, and coating with inert materials. **c** Schematic illustration of engineering catalyst-electrolyte interface via tuning the surface-wetting state, promoting mass transport, altering adsorbed ions, and local electrolyte environment

The catalytic processes are closely correlated to the physicochemical properties of catalyst surfaces, which directly affect the adsorption and desorption of reaction species [43]. Through years of efforts, various strategies, including facet engineering, defect engineering, chemical doping, and surface functionalization, have been developed to enhance the electrocatalytic performance of catalysts (Fig. 3a) [44]. These promoting strategies have a considerable effect on modulating the electronic structure of catalytic material for favorable reaction kinetics [43]. For example, the chemical doping- and defect-induced exotic changes in the local chemical environment have been regarded to manipulate the surface electronic states, such as increasing the density of states (DOS) near the Fermi level, narrowing the bandgap, and shifting the *d*-band center position of metal catalysts [45]. Thus, establishing the electronic structure–property relationship of catalyst materials and exploring how the electronic structure affects catalytic performances are important for the design and synthesis of efficient electrocatalysts.

Apart from the electronic configuration, many catalytic reactions are structure sensitive, meaning that the reaction rate depends on the geometrical structure of the catalyst surface [46]. The selectivity can also be controlled by geometric or ensemble effects, which are associated with the geometric arrangement of atoms in the catalyst surface and adsorption configurations of intermediates [9]. For instance, the end-on O_2_ adsorption configuration can be regulated by eliminating the accessible active metal ensembles. Such site isolation can also be realized by atomization, alloying with a secondary metal, and coating with inert materials (Fig. 3b). By these means, the likelihood of decomposition or further electrochemical reduction of H_2_O_2_ can also be minimized.

Since electrocatalytic ORR occurs at the solid–liquid-gas triple-phase among O_2_, solid catalysts, and electrolyte solutions, engineering the reaction interface is essential for boosting the overall 2e^–^-ORR performance. The catalysts and electrodes should allow rapid mass transport of reactants and products for a high production rate. Tuning the porosity (pore connectivity, length, and pore size distribution) and the surface-wetting state of electrode surfaces would be the most straightforward strategy. At the same time, the electrolyte provides proton sources and enables the transport of charged ion species. The local electrolyte environment (e.g., pH value and the cation/anion compositions) dramatically affects practical catalytic performances via multiple mechanisms (Fig. 3c). Therefore, proper modification of the electrolyte to tailor the local reaction environment provides a new opportunity to optimize the overall performance for H_2_O_2_ production.

## Designs of Electrocatalysts for 2e^–^-ORR

The selective formation of H_2_O_2_ through the 2e^–^-ORR is more thermodynamically unfavorable than the 4e^–^ counterpart toward H_2_O [47]. Suitable catalyst materials play a key role in controlling the reaction kinetics and reaction route. Overall, a promising electrocatalyst for electrochemical H_2_O_2_ production should possess three key indicators for catalytic performances, including activity, selectivity, and stability [11]. Based on computational screening and experimental investigations, many prospective electrocatalysts have been suggested, including noble metals and their alloys, carbon-based materials, single-atom catalysts (SACs), organic molecules, etc. In this section, recent advances in theoretical and experimental studies for different categories of 2e^–^-ORR electrocatalysts construct a comprehensive roadmap of engineering strategies in the practical design of electrocatalysts.

### Noble Metals and Alloys

#### Regulating the Atomic Arrangement

Au has been intensively investigated and identified as a typical 2e^–^-ORR electrocatalyst [48, 49]. According to the activity volcano plot, Au is on the weak binding side with the limiting step for forming *OOH [28]. However, the kinetics and the reaction mechanism vary on the Au surfaces, depending on the crystallographic orientation. For instance, Adzic et al. reported the 4e^–^ reduction of O_2_ to H_2_O on the Au(100) surface, while 2e^–^ reduction to H_2_O_2_ on Au(111) and Au(110) surfaces [50].

**Fig. 4:**
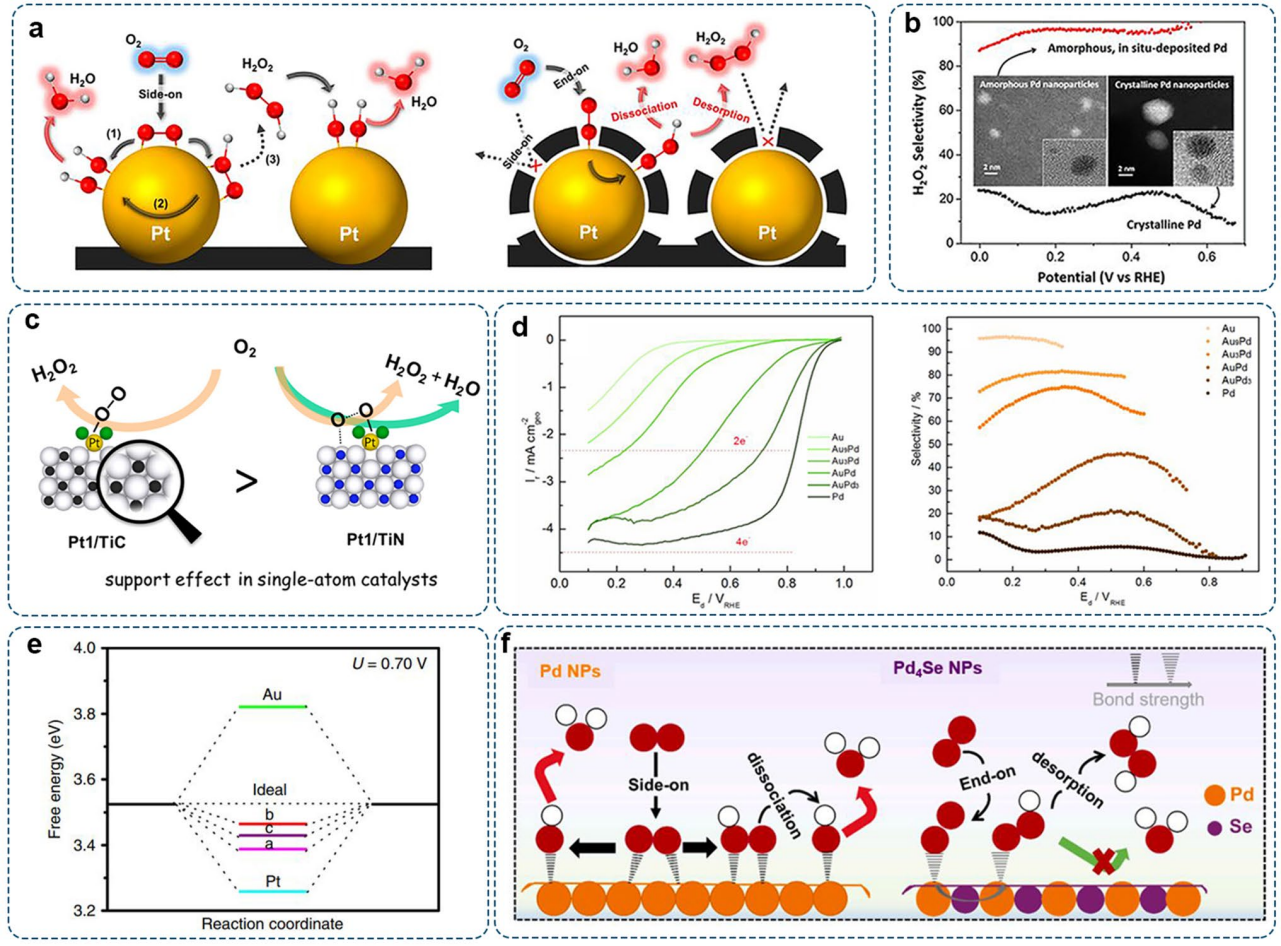
Representative noble metal electrocatalysts for 2e^–^-ORR: **a** Schematic of the pristine Pt catalyst and Pt coated by carbon layer. Reproduced with permission [52]. Copyright 2014, American Chemical Society. **b** H_2_O_2_ selectivity on amorphous and crystalline Pd nanoparticles. Reproduced with permission [53]. Copyright 2019, American Chemical Society. **c** Schematic illustration of the support effect in single-atom Pt catalysts. Reproduced with permission [54]. Copyright 2017, American Chemical Society. **d** RRDE results of Au–Pd catalysts with different compositions in O_2_-saturated 0.1 M HClO_4_ and corresponding H_2_O_2_ selectivity. Reproduced with permission [55]. Copyright 2017, Elsevier. **e** Free-energy diagram of Au, Pt, and PtP_2_ for O_2_-to-H_2_O_2_ at 0.70 V (a, b and c represent the top, bridge, and hollow sites of PtP_2_ (111) surface). Reproduced with permission [56]. Copyright 2017, Springer Nature. **f** Promoted 2e^–^-ORR pathway on Pd_4_Se via geometric and electronic effects. Reproduced with permission [57]. Copyright 2021, Elsevier

For Pt and Pd with the strong *OOH binding energy, the 4e^–^-pathway is preferred over the 2e^–^-pathway [9]. However, the reaction pathway can be altered by manipulating the surface geometry and accessibility of active sites [51]. The elimination of Pt–Pt ensembles via coating with amorphous carbon layers has been demonstrated to induce the end-on adsorption mode of O_2_, resulting in promoted 2e^–^-ORR selectivity compared with pristine Pt/C (Fig. 4a) [52]. Amorphous Pd nanoparticles were synthesized through the in-situ electrochemical deposition of Pd^2+^ ions and displayed higher selectivity (above 95%) than crystalline Pd-based catalysts, which could be attributed to the decreased quantity of adjacent Pd atoms in the amorphous structure (Fig. 4b) [53].

The ORR over the catalyst nanoparticles is a typical structure-sensitive reaction [58]. Jirkovský and co-workers reported that the selectivity for 2e^–^-ORR was higher when particle sizes of Au were below 6 nm [48]. Pt nanoparticles were observed to have enhanced H_2_O_2_ productivity when the nanoparticle size decreased [59]. Such particle size-dependent behavior is associated with the oxygen-binding energies on the different active sites (e.g., edges, steps, and defects) accessible on particles with various sizes [60]. By reducing the loading amount of noble metal and enlarging interparticle distance, the electrocatalytic activity and selectivity showed an increase due to a lower amount of neighboring active sites, thus hindering the breakup of O–O bond [61–63]. Moreover, with small particle size and dilute noble-metal content, it would be easier for generated H_2_O_2_ to escape from the catalyst surface and diffuse into the bulk electrolyte, thus avoiding further decomposition [64].

#### Atomization Engineering

As the theoretically ultimate size limit, single-atom catalysts (SACs), in which the finely dispersed single-metal atoms over the substrates produce a similar coordination environment for each reaction center, can afford desired reaction selectivity and activity [65, 66]. Compared to their bulk metal counterparts, the lack of contiguous metal sites in SACs would prevent the chemical dissociative mechanism, thus limiting the competitive 4e^–^-route. Meanwhile, the decomposition of H_2_O_2_ on SACs is also inhibited since the dissociative chemical re-absorption (Eqs. (5) or (7)) prefers to occur on two neighbouring adsorption sites [28]. It has been reported that the ORR follows the 2e^–^ pathway on Pt-SACs, producing H_2_O_2_ as the end product [64, 67]. Other examples include atomically dispersed Au, Ru, Rh, Pd, Os, and Ir on carbon or non-carbon substrates with enhanced 2e^–^ selectivity [68–70]. Therefore, the metal site isolation can effectively tune the selectivity for target reactions.

The host materials also play a significant role in affecting the catalytic properties of SACs. Atomically dispersed Pt on TiC showed favorable adsorption energy and energy profiles toward the 2e^–^-ORR, resulting in superior activity and selectivity [54]. In contrast, oxygen species had a strong affinity to Pt on TiN, resulting in sluggish activity (Fig. 4c). Li’s group developed a redox-based ion-exchange approach to anchor a high concentration of atomically dispersed Pt (24.8 at%) on the hollow CuS_x_ substrate [71]. The strong interaction of Pt–S ensured the well-isolated Pt atoms with high concentrations but without aggregation. Meanwhile, the hollow and porous structure of the CuS_x_ substrate benefited the mass transport and the exposure of more reactive sites for the catalytic process. The resultant h-Pt_1_-CuS_x_ displayed high selectivity of 92%‒96% in the potential range of 0.05‒0.7 V (vs. RHE). The electrochemical device was constructed with the h-Pt_1_-CuS_x_ catalyst to synthesize H_2_O_2_ from H_2_ and O_2_. Impressively, it generated a high yield of 546 mol kg_cat_^−1^ h^−1^.

#### Multicomponent Alloying

Metals such as Hg and Au exhibit high 2e^–^ selectivity due to their weak oxygen binding [17]. Meanwhile, they also exhibit a low reaction rate and high overpotential. Constructing metal alloys is a promising strategy to promote activity and maintain high selectivity simultaneously. The interaction with adsorbed intermediates can be finely tuned due to changes in the *d*-band center, orbital hybridization, and charge distribution of constitutive metals [46, 72]. Meanwhile, the geometric structure is changed because incorporated foreign elements disrupt the monometallic ensembles of contiguous metal atoms [46]. As a result, the binding strength and configuration of adsorbed intermediates will be altered to a suitable level compared to the pristine constitutive metals. Siahrostami et al. screened over 30 alloy catalysts by using DFT calculations [9]. The PtHg_4_ alloy was predicted to exhibit optimal binding energy for *OOH with the smallest thermodynamic overpotential. They experimentally synthesized the Pt@Hg/C catalyst consisting of the Pt core and Pt–Hg shell. Electrochemical measurements confirmed that Pt@Hg/C catalyst was highly active and selective for electrochemically reducing O_2_ to H_2_O_2_. Specifically, the selectivity was up to 96%, and the mass activity was 26 ± 4 A g^−1^_(Pt)_ at the overpotential of 50 mV. They further studied trends for H_2_O_2_ production on different metal surfaces modified with Hg [73]. They found that Pt–Hg and Ag–Hg exhibited an order of magnitude improvement over metal Au, and the activity of Pd–Hg was two orders of magnitude higher. It was proposed that the bulk alloys consisting of the isolated active sites surrounded by inert elements could boost activity and preserve selectivity toward the desired H_2_O_2_ product by not allowing O−O dissociation.

Bimetallic Pt–Au [74]. and Pd–Au [75] alloys have displayed a high H_2_O_2_ yield of > 90% in acid media. Freakley et al. studied the impact of composition on activity and selectivity by systematically investigating the electrocatalytic performance of a set of Au–Pd nanoparticles with different Au: Pd molar ratios [55]. They found that the ORR pathway changed from 4e^–^ to 2e^–^ with increasing Pd content (Fig. 4d). Therefore, regulating alloying components and composition is crucial for optimizing the 2e^–^ selectivity. Considering the difference in atomic radius and lattice constant of constitutive materials, the induced geometrical strain is an important factor in influencing the catalytic properties of alloy catalysts. For instance, to reduce the lattice distortion between Au and Ni, Pt was introduced as the transition layer [76]. The altered lattice strain promoted the 2e^–^ selectivity up to 95%.

Additionally, alloying noble metals with non-metal elements such as C, P, and S shows improved 2e^–^-ORR performances. Li et al. reported the monodisperse colloidal platinum diphosphide nanocrystals (PtP_2_ NCs), which displayed a maximum selectivity of 98.5% at 0.27 V (vs. RHE) [56]. The incorporation of P not only changed the electron density of Pt but also separated the adjacent Pt–Pt ensemble. As a result, end-on adsorption of *OOH was dominated on the PtP_2_ surface with weaker binding strength than pure Pt (Fig. 4e). In another work, Pd atoms isolated by semi-metal Se would favor the end-on adsorption of O_2_ [57]. A strong p-d repulsive correlation shifted the Pd-4d band towards the electron-depleting center, thus facilitating *OOH stabilization for H_2_O_2_ generation (Fig. 4f).

Although noble metals and their alloys present good activity and selectivity in acidic media, the imperative high cost and scarcity of noble metal-based materials might constrain their large-scale applications. To this end, tremendous efforts have been devoted to developing noble metal-free catalysts as alternatives, which will be discussed in the following sections.

### Non-Precious Transition Metal Catalysts (NPTMCs)

Transition metal-based materials with low cost and high abundance are attractive alternatives to substitute noble metal-based materials [77]. However, the dissociation of O_2_ and disproportionation of H_2_O_2_ favorably occur on the transition metal surface due to closely packed active sites [78]. Through proper material engineering, including tuning crystal structures, controlling the morphology, engineering exposed phases, inducing vacancies, regulating cations/anions, and integrating with carbon hosts, favorable catalytic surfaces with suitable *OOH binding can be obtained on transition metal-based catalysts for 2e^–^-ORR.

#### Controlling Morphology of NPTMCs

Jin’s group reported the CoS_2_ [35]. and CoSe_2_ [79]. catalysts, in which the neighboring Co sites were separated by chalcogenide anions, thus suppressing the 4e^–^-process. Moreover, the test results showed that CoS_2_ and CoSe_2_ were chemically stable in acid media, making them practical candidates for the electrochemical synthesis of H_2_O_2_. Their studies inspired the screening of transition metal dichalcogenides, such as NiS_2_ and Ni_2_Mo_6_S_8_, as 2e^–^-ORR catalysts [80, 81].

**Fig. 5:**
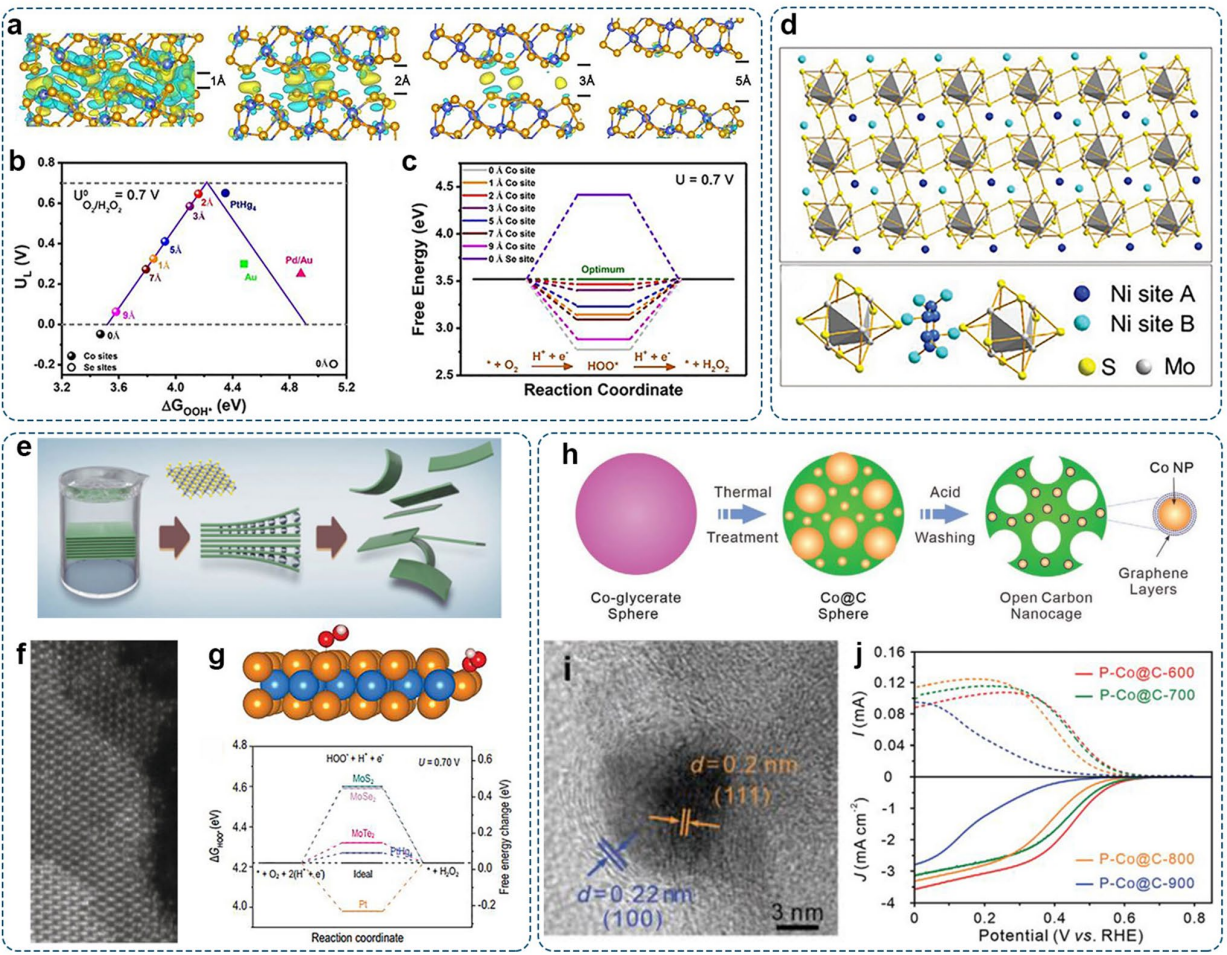
Representative NPTMCs for the 2e^–^-ORR: **a** Differential charge densities of CoSe_2_ with various interlayer distances (yellow and cyan contours represent charge accumulations and depressions, respectively). **b** Calculated volcano plot for the 2e^–^-ORR plotted as a function of ΔG_*OOH_. **c** Free energy diagram for O_2_-to-H_2_O_2_ at 0.70 V. Reproduced with permission [83]. Copyright 2021, Wiley–VCH. **d** Schematic illustration of the crystal structure of Ni_2_Mo_6_S_8_. Reproduced with permission [81]. Copyright 2021, Wiley–VCH. **e** Schematic illustration of liquid-phase exfoliation method for producing MoTe_2_ nanoflakes. **f** Scanning transmission electron microscopy image (STEM) of the MoTe_2_ edge structure. **g** The optimized structure of *OOH adsorbed on the basal plane or edge site of MoTe_2_ (blue, orange, red, and white spheres represent Mo, Te, O, and H atoms, respectively) and calculated free energy diagram for O_2_-to-H_2_O_2_. Reproduced with permission [84]. Copyright 2020, Oxford University Press. **h** Schematic illustration of the preparation of Co NPs-embedded open carbon nanocage. **i** High-resolution transmission electron microscopy (HRTEM) images of P-Co@C-700 nanocages. **j** LSV curves with ring current and disk current density of P-Co@C catalysts in 0.1 M HClO_4_. Reproduced with permission [85]. Copyright 2022, Wiley–VCH

The electrocatalytic properties closely correlate with the morphology of the catalyst. By reducing the size and dimension, the exposure of under-coordinated surfaces and active sites can be maximized for enhanced performance. Ji et al. tuned the morphology of CoSe_2_ from three-dimension (3D) to two-dimension (2D) by adjusting the wettability of the growth substrate [82]. The ultrathin CoSe_2_ nanosheets grown on the hydrophilic carbon cloth nanosheets demonstrated selectivity of 92% towards the 2e^–^-ORR, which was significantly higher than that of 3D nanostructures grown on hydrophobic carbon cloth (72%). For 2D materials, the interlayer spacing of adjacent layers significantly affects the electrochemical performances. Gu et al. applied an H^+^-exchange method to replace the protonated diethylenetriamine (DETA) molecules that were sandwiched between CoSe_2_ layers [83]. Consequently, the interlayer spacing of CoSe_2_ was reduced from −5.3 Å to −2 Å, without extra structure changes. The altered interlayer coupling induced the changes in the electronic states of CoSe_2_ layers, hence affecting the adsorption energy for *OOH (Fig. 5a–c).

In general, morphology engineering can be broadly applied to transition metal materials to alter the exposure of active sites and electronic structure for regulating the reaction route and product selectivity.

#### Phase Engineering of NPTMCs

The electronic states and physicochemical properties generally vary on different crystallographic planes due to different atomic coordination and densities [86]. This explains why the same crystalline materials with different exposed facets usually display different catalytic behaviors. Phase engineering is an essential electronic-structure modulation strategy for promoting 2e^–^-ORR performances via optimizing the atomic configuration. Cheng’s group reported the Chevrel phase chalcogenide Ni_2_Mo_6_S_8_ for selectively reducing O_2_ to H_2_O_2_ [81]. The Chevrel phase exhibited a versatile crystal structure with 3D channels from interconnected Mo_6_S_8_ clusters, each having a Mo_6_ octahedron surrounded by an S_8_ cube. Due to the resulting spatial confinement effect, atomic Ni active sites were isolated (Fig. 5d). The Ni_2_Mo_6_S_8_ catalyst provided highly efficient H_2_O_2_ production with > 90% selectivity within a wide potential range (0–0.6 V vs. RHE) and a high-yield rate of ~ 90 mmol H_2_O_2_ g_cat_^−1^ h^−1^. Li’s group synthesized 2H-phase molybdenum telluride (MoTe_2_) nanoflakes from the bulk powder via ultrasonication-assisted liquid-phase exfoliation (Fig. 5e) [84]. The exfoliation-induced exposure of edges with uncoordinated Mo and Te bonds would significantly promote catalytic activity and selectivity (Fig. 5f). The H_2_O_2_ selectivity of MoTe_2_ nanoflakes was up to ∼93%, and the mass activity was 27 A g^−1^ at 0.4 V (vs. RHE) in 0.5 M H_2_SO_4_. Theoretical calculations further revealed that active sites at the zigzag edges exhibited favorable binding toward *OOH (Fig. 5g).

#### Inducing Vacancies in NPTMCs

The presence of vacancies in the lattice plane can alter the surface chemistry of transition metal-based materials due to the decreased coordination number of surrounding atoms, which is beneficial for enhancing charge transfer and reaction kinetics [87]. Particularly, the distance between the two nearby active sites can be prolonged by creating cation vacancies on the host materials, thus optimizing the *OOH adsorption. Attributed to these electronic and geometric advantages, Ni cationic vacancies (V_Ni_)-enriched nickel phosphide (Ni_2−x_P-V_Ni_) showed an excellent 2e^–^-ORR performance with H_2_O_2_ molar fraction above 95% and Faradaic efficiency above 90% in all pH conditions [88].

Oxygen vacancy is a typical type of anion vacancy on transition metal oxides. Promoted 2e^–^-ORR catalytic activity has been reported on oxygen-deficient TiO_2-x_ and Co_3_O_4-x_ due to modified electronic structures [89–91]. Besides the electronic effects, the configuration of O_2_ adsorption on transition metal oxides can be regulated by oxygen vacancies. Zou et al. simultaneously conducted phase and defect engineering on α-Fe_2_O_3_ [92]. The [001] facet of α-Fe_2_O_3_ with oxygen termination presented weak O_2_ adsorption. When the oxygen vacancy was created on the [001] facet, the O_2_ molecule could occupy the vacant site via the “end-on” configuration, in which one oxygen atom filled into the vacant site. As discussed early, such an adsorption configuration could avoid O–O cleavage and afford high selectivity for H_2_O_2_ production. The synthesized oxygen defective α-Fe_2_O_3_ single crystals with exposed [001] facets achieved high selectivity of > 90%, > 88%, and > 95% in weakly acidic, neutral, and alkaline electrolytes, respectively.

#### Cation/Anion Regulation in NPTMCs

For transition metal materials, the intrinsic activity of active metal cations can be effectively manipulated by substituting metal cations. The incorporated atoms may serve as a barrier to isolate the active site. Moreover, the different atom radii would change atomic arrangements, electronic states, and surface chemistry, thus bringing fascinating physicochemical properties for electrochemical reactions. For example, Ni was incorporated to substitute part of tetrahedral and octahedral Co sites in the CuCo_2_S_4_ thiospinel catalyst [93]. In comparison with pristine CuCo_2_S_4_, the 2e^–^-selectivity was enhanced on resultant CuCo_2−x_Ni_x_S_4_ (0 ≤ x ≤ 1.2) catalysts (> 60%). Meanwhile, the overpotential was also reduced along with the increase of Ni content, demonstrating an enhanced activity. To alter the *d*-band center position and adjust the intermediate binding strength, Chen and co-workers incorporated 3d transition metal atoms into columbites (MNb_2_O_6_, M = Mn, Fe, Co, Ni, and Cu) using polyoxoniobate (K_7_HNb_6_O_19_·13H_2_O) and various divalent metal cations by a hydrothermal method [94]. The optimal NiNb_2_O_6_ delivered an H_2_O_2_ selectivity of 96% in alkaline media, and the production rate was up to 1 mol H_2_O_2_ g_cat_^−1^ h^−1^ in an H-shaped electrolyzer. Operando FTIR analysis suggested Nb sites were more active than Ni, thus serving as the catalytic sites for H_2_O_2_ synthesis. As indicated by DFT calculations, the *d*-band center of catalytically active surface Nb atoms was mediated by doped 3d transition metals, hence altering its catalytic activity for electrochemical H_2_O_2_ synthesis.

Similarly, the change of surrounding anions can generate another substantial effect on ORR activity and selectivity via modulating the electronic structure of active sites. For instance, the vulcanized NiP_4_Mo_6_ polyoxometalate (s-NiP_4_Mo_6_) presented the 2e^–^-ORR pathway with selectivity between 98.3 and 96.5%, while the 4e^–^-process was presented on the pristine NiP_4_Mo_6_ [95]. DFT calculations suggested that the adsorption of *OOH was weakened when one μ_2_-O was replaced by the low electro-negativity S^2−^ anion.

#### Carbonaceous Integration with NPTMCs

The pristine transition metal compounds generally exhibit poor conductivity and suffer from easy aggregation, which might impede electron transfer and mass transport during the electrochemical process. Compositing inorganic transition metal-based electrocatalysts with high-conductive carbon materials can effectively enhance the conductivity and improve the dispersion of metal moieties. Moreover, the interfacial interaction between metal nanoparticles and carbon supports can induce the electronic structure reconfiguration and shift the valence states of metal nanoparticles, thus further enhancing the catalytic performance. This design concept has been successfully applied in 4e^–^-ORR, resulting in numerous composite electrocatalysts with high activity and durability [96, 97]. This strategy is also applicable for designing 2e^–^-ORR catalysts. The resultant hybrid catalysts would further promote catalytic activity and selectivity via altered electronic structure and elaborated architecture. Yu et al. fabricated mesoporous open carbon nanocages with embedded Co nanoparticles by using Co-glycerate spheres as the template (Fig. 5h) [85]. Co nanoparticles were coated with graphitic carbon layers in the resultant P-Co@C sample (Fig. 5i). The optimal P-Co@C-700 sample exhibited excellent 2e^–^-ORR performance with selectivity up to 94% in 0.1 M HClO_4_ (Fig. 5j).

Suppressing H_2_O_2_ decomposition or further reduction while achieving high activity and selectivity is important for electrocatalyst optimization, especially for the metal-based catalysts that are kinetically favorable for peroxide decomposition. To overcome this impediment, Li et al. reported a steric hindrance and layered structure induction strategy for fabricating nanohybrid electrocatalysts [78]. Ni-LDH ultrathin chips in-situ inlaid on carbon nanosheets (Ni-LDH C/CNSs) for 2e^–^-ORR. DFT calculations suggested that increased Ni edge sites with more deficient O atom coordination were active sites. The modified carbon nanosheets as the steric hindrance could effectively suppress the H_2_O_2_ decomposition and avoid the O−O bond breaking. Benefiting from the synergistic effect of CNSs and Ni-LDH chips, a low overpotential of about 45 mV and high selectivity with a Faradic efficiency of up to 95% were achieved on Ni-LDH C/CNSs.

It is essential to bear in mind that the carbon matrix in the composites may also serve as a co-catalyst and contribute to H_2_O_2_ production. The catalytic contribution of carbon materials will be explicitly discussed in Sect. 3.3. In the hybrid composite, some metallic species are encapsulated by graphitic carbon shells. Such a core–shell structure would prevent the metallic species from directly interacting with reactants. Although the resistance against electrolyte leaching is enhanced, it also raises the question about the role of metal entities in the carbon matrix [98]. Previous studies suggested that encapsulated metal entities could modulate the electronic structure of coated carbon skeleton via charge coupling [85, 99]. Reaction intermediates are absorbed on activated carbon layers. However, some studies have pointed out that electron penetration was limited to four carbon layers [100]. One may argue that reactants could still penetrate through the pores and voids of the carbon framework and reach the activated layers. It has been proposed that the encapsulated metal residues on the carbon matrix surface might lead to the formation of atomic metal sites, which participate in ORR as active sites [101–103]. Thus, the specific roles of metallic species and carbon shells in enhancing catalytic performance still need further investigation.

### Metal-Free Carbon Materials

The earth-abundance, low cost, outstanding stability, electroconductivity, excellent mechanical properties, and versatile structures of carbon materials make them a front runner in electrocatalysis applications [104–106]. Early studies suggest that the pristine carbon materials are inactive for H_2_O_2_ production [3, 107]. The activity and selectivity are relatively low due to the unsuitable electronic structure for *OOH binding [108]. Thus, it is essential to tailor the carbon surfaces so that the electronic and physical structures can be rationally modulated, resulting in enhanced activity and selectivity for electrocatalytic reactions. The state-of-the-art strategies for the atomic and structural design of carbon-based 2e^–^-ORR catalysts include doping heteroatoms, modifying with oxygen functional groups, creating defects, and engineering porosity. In this section, recent progress in metal-free carbon materials for electrocatalysis H_2_O_2_ is divided into several categories according to the applied design strategy and will be discussed separately.

#### Heteroatom-Doped Carbon Catalysts

Doping heteroatoms (e.g., N, B, O, F, S, P) into the carbon framework is an effective strategy for tuning carbon catalysts' physical and chemical properties [109, 110]. The promoted electrocatalytic activity originates from electroneutrality breaking and electron transfer induced by the size and electronegativity difference of incorporated heteroatoms [111]. The electron configuration of the adjacent carbon can be altered, and the charge/spin distribution on the pristine *sp*^*2*^-hybridized carbon structure can be effectively modified, leading to optimized intermediate chemisorption and consequently improving the intrinsic activity.

**Fig. 6:**
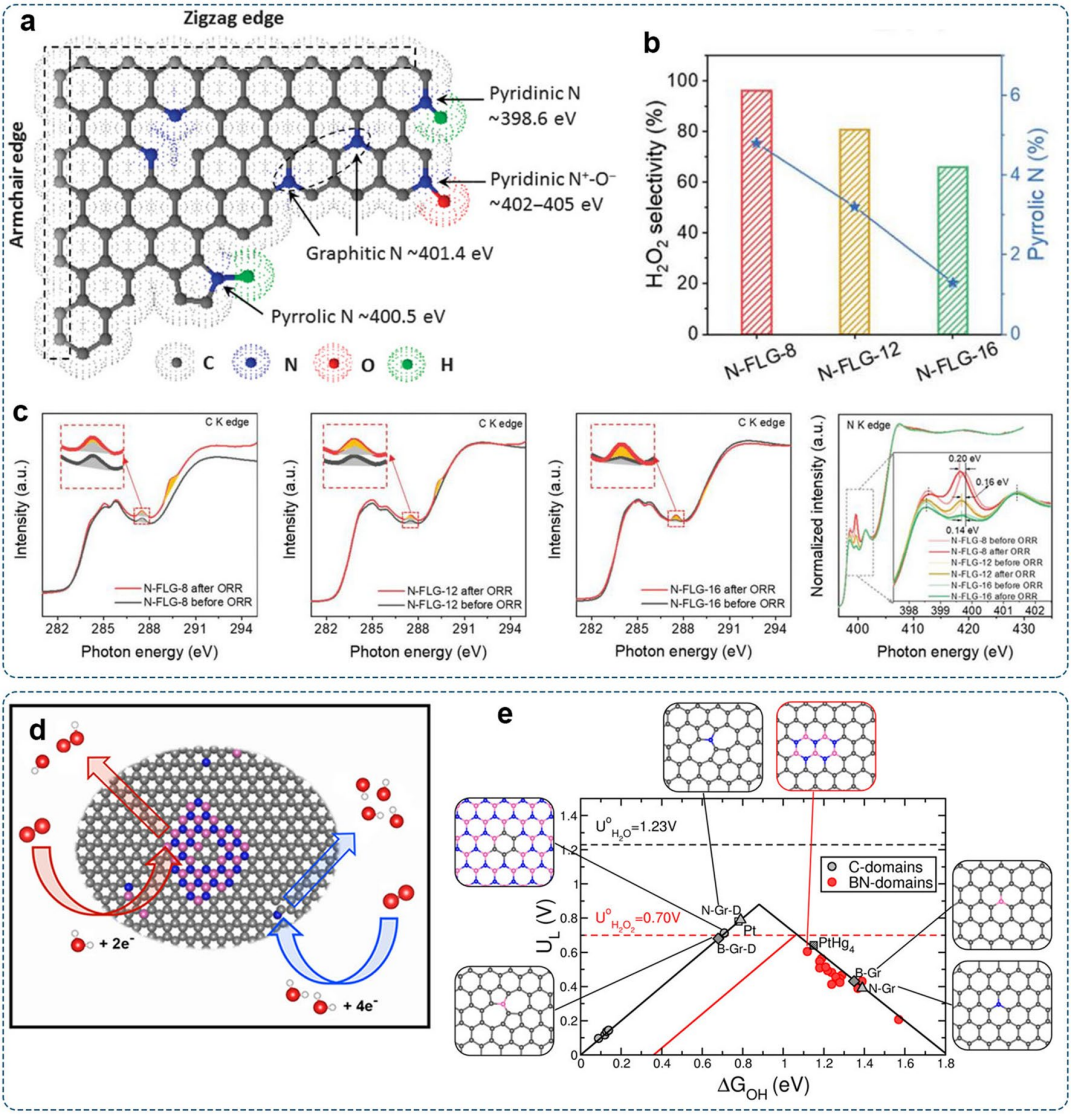
Heteroatom-doped carbon catalysts:** a** Scheme illustration of N-doping configurations in graphitic carbon and corresponding X-ray photoelectron spectroscopy (XPS) binding energies. Reproduced with permission [106]. Copyright 2015, AAAS. **b** Relationship between H_2_O_2_ selectivity and atomic content of pyrrolic-N. **c** Elucidation of the activity origin for H_2_O_2_ generation based on C and N K-edge XANES spectra. Reproduced with permission [113]. Copyright 2021, Wiley–VCH. **d** Schematic illustration of B,N co-doped carbon material for H_2_O_2_ production. **e** Configuration models of B,N-codoped carbon, and calculated volcano plot for the 2e^–^-ORR plotted as a function of ΔG_*OOH_. Reproduced with permission [115]. Copyright 2018, American Chemical Society

N-doping has been widely applied to improve the ORR performances of carbon-based materials. The N atom could be doped into several locations within the graphite plane, leading to multiple configurations, such as pyridinic N, pyrrolic N, graphitic N, or oxidized N (Fig. 6a) [106]. The catalytic properties are highly dependent on the N-doping configurations, which affect adsorption properties toward oxygen-containing intermediates [17]. The pyridinic N is known to favor the 4e^–^-pathway [112]. It can provide delocalized lone pair electrons to enhance the electron-donating capability of the carbon matrix [113, 114]. The O−O bond is weakened due to the charge transfer from the π orbital to the antibonding orbitals in O_2_, and eventually dissociated into *O and *OH. In contrast, the pyrrolic N has shown a positive correlation with H_2_O_2_ selectivity (Fig. 6b) [113]. The variable adsorption profiles of *OOH and *O intermediates on C K-edge X-ray absorption near-edge structure (XANES) spectra and the negative shifts of the pyrrolic N peak on N K-edge XANES spectra of N-rich few-layered graphene (N-FLG) catalysts verified the essential role of the pyrrolic N in the 2e^–^-ORR process (Fig. 6c) [113].

Other than N-doping, B-[116], O-[117, 118], F-[119], S-[120], and P-doped [121], carbon materials also demonstrated the enhanced capability for electrosynthesis of H_2_O_2_ due to induced charge redistribution. It should be noted that most studies regard the C atom adjacent to the N atom as the adsorption site, while exceptions may exist in some other heteroatom-doped carbon materials. A recent study by Wang and co-workers investigated the 2e^–^-ORR activity on different heteroatom-doped (B-, N-, S-, P-) carbon materials [116]. For B-C and P–C, the most favorable adsorption sites for *OOH were B and P instead of the neighboring C.

Dual- and triple-doping has also been applied to improve electrocatalytic activities of carbon-based catalysts toward higher reaction efficiency [122, 123]. Bao’s group prepared B, N co-doped carbon materials with hexagonal boron nitride (*h*-BN) in the carbon lattice (Fig. 6d) [115]. Such hybrid structures yielded high activity and selectivity (60%-85%) toward the 2e^–^-ORR to H_2_O_2_. DFT calculations showed that the interface between *h*-BN domains and graphene exhibited favorable catalytic behavior toward 2e^–^-ORR (Fig. 6e). Similar examples are demonstrated on N,F co-doped carbon nanocages [124], N,S co-doped mesoporous carbon [125], and N,O co-doped carbon materials [126, 127].

#### Oxygen Functionalization of Carbon Catalysts

Oxidized carbon materials are emerging as promising candidates for the 2e^–^-ORR [30]. Various oxygen functional groups (e.g., –COOH, C–O–C, C=O) have been incorporated into the carbon matrix and presented the potential to optimize the 2e^–^-ORR performance. Cui and co-workers deliberately modified carbon nanotubes with oxygen functional groups via concentrated nitrate acid treatment [128]. After oxidizing, C–O and C=O containing functional groups were found on the catalyst surface. The RRDE tests showed that oxidized carbon nanotubes (O-CNTs) presented remarkably enhanced activity and selectivity compared to pristine CNTs, verifying the significant contribution of oxygen functional groups to H_2_O_2_ generation.

McCloskey and co-workers synthesized a metal-free carbon catalyst through annealing a few-layered mild reduction of graphene oxide (GO) at 600 °C [32]. The obtained *F*-mrGO (600) catalyst achieved a low overpotential (< 10 mV) with nearly 100% selectivity and excellent stability in 0.1 M KOH. Multiple characterization analyses suggested that the epoxy or ether groups along the basal plane or at the sheet edges were responsible for the high H_2_O_2_ formation activity. This conclusion was further supported by theoretical investigations, which showed that graphene edges with epoxy and ring ether groups exhibited optimal 2e^–^-ORR activity [129].

**Fig. 7:**
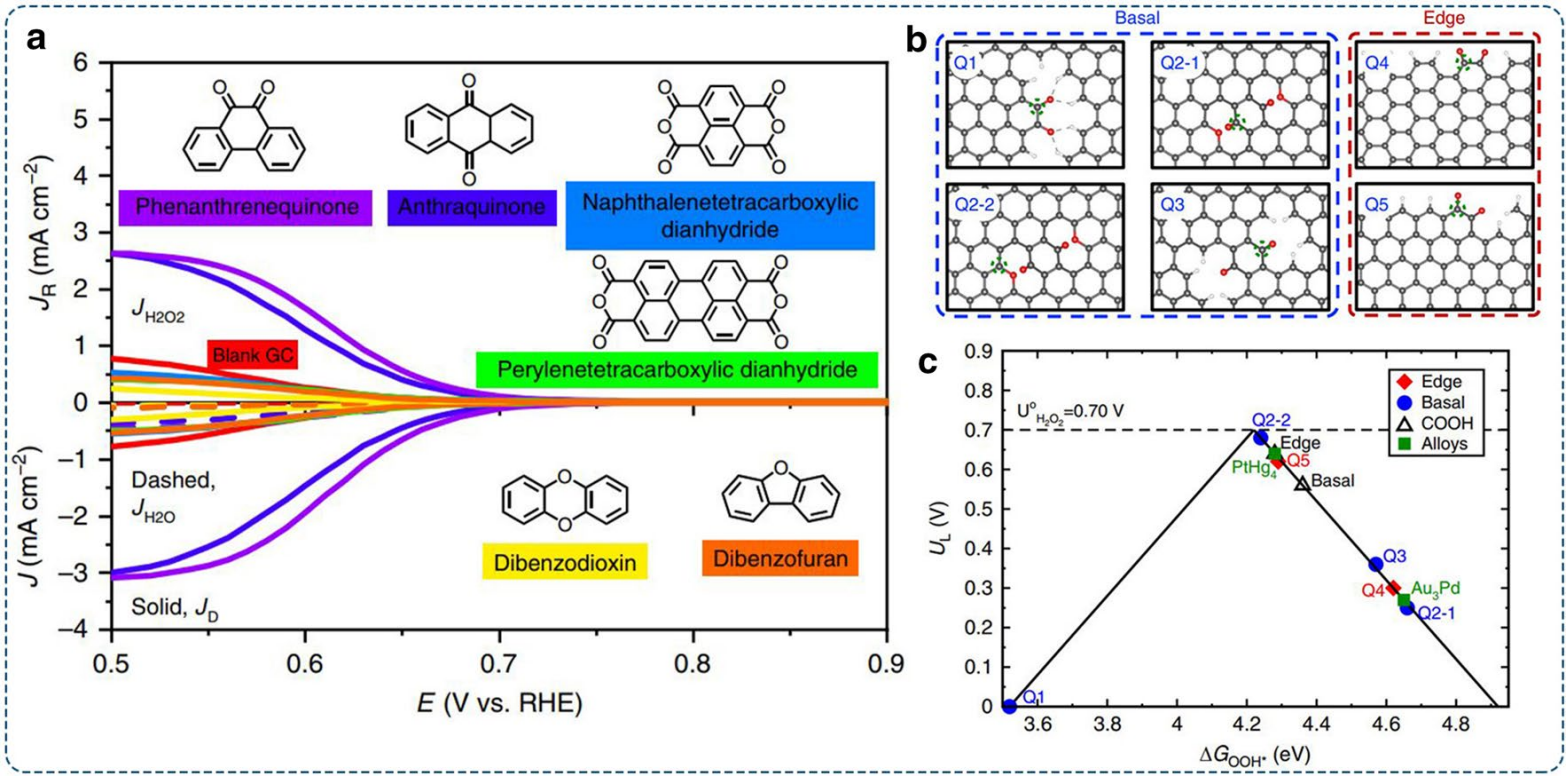
Oxidized carbon electrocatalysts: **a** LSV curves of standalone molecules showing the disk and ring currents. **b** The atomic structures of the examined oxygen functional groups. The corresponding active sites are marked with a dashed green circle. **c** The activity volcano plot at the thermodynamic equilibrium potential of 0.70 V. Reproduced with permission [36]. Copyright 2020, Springer Nature

The cleavage of *sp*^*2*^ C–C bonds and incorporation of oxygen functional groups may lead to multi-components. To identify the most active one, Jong-Beom et al. decorated the dangled edges of graphitic materials with ether, carboxyl, and quinone groups via oxidation with CO_2_ or diluted O_2_ [36]. The quinone-enriched sample (GNPC = O,1) had the highest activity with the H_2_O_2_ yield ratio of 97.8% at 0.75 V in alkaline media, suggesting that the quinone functional group was the most active. The result was further verified by standalone molecular chemistry, in which only the phenanthrenequinone and anthraquinone with the quinone group showed 2e^–^-ORR activity (Fig. 7a). The activity trends of different possible quinone functional groups in the edge and basal plane of the carbon nanostructure were examined by DFT calculations. The configuration with quinone groups on the basal plane (Q-basal 2–2) presented higher activity (Fig. 7b–c). But considering the easier formation of quinone functional groups on the edges, the quinone-edge structure (Q-edge 5) was identified as the potential active site for selective 2e^–^-ORR.

Besides high activity and selectivity, another advantage of oxidized carbon materials is their simple and feasible preparation procedures, which can be conducted under mild conditions. For example, Chen et al. synthesized reduced graphene oxide via KOH aqueous solution treatment (rGO_-KOH_) [130]. The resultant rGO_-KOH_ exhibited abundant C–O–C groups and a large electrochemically active surface area. The selectivity of ~ 100% was achieved on rGO_-KOH_ for electrochemical H_2_O_2_ production. Li et al. developed a simple, fast, and readily scalable means to develop high-performance electrocatalysts involving the microwave treatment of ordered mesoporous carbon (CMK3) and subsequent liquid nitrogen freezing [131]. The whole procedure only costs one minute. The C=O bond in pristine carbon materials could be broken to form C–OH or C–O–C during the rapid microwave treatment. The liquid nitrogen treatment could prevent the further oxidation of produced C–OH or C–O–C. The resultant catalyst displayed significantly enhanced performance with a selectivity of ~ 90% and Faradaic efficiency of ~ 95% in the alkaline solution. The productivity of H_2_O_2_ could reach 2,476 mmol g_cat_^−1^ h^−1^ at 0.3 V (vs. RHE). Moreover, Roman et al. reported that graphene edge sites could readily be functionalized with C=O and –OH groups even under ORR operations [132].

#### Defect Engineering in Carbon Catalysts

The defects formed at both edges and bulk domains of carbon-based materials are essential structure properties that affect the catalytic performance [133]. The defects have been considered to change the local electronic structure of carbon frameworks, thus affecting the binding strength of the intermediate [134, 135]. Therefore, deliberately manipulating defects in carbon materials is an effective method to enhance electrocatalytic performance.

**Fig. 8:**
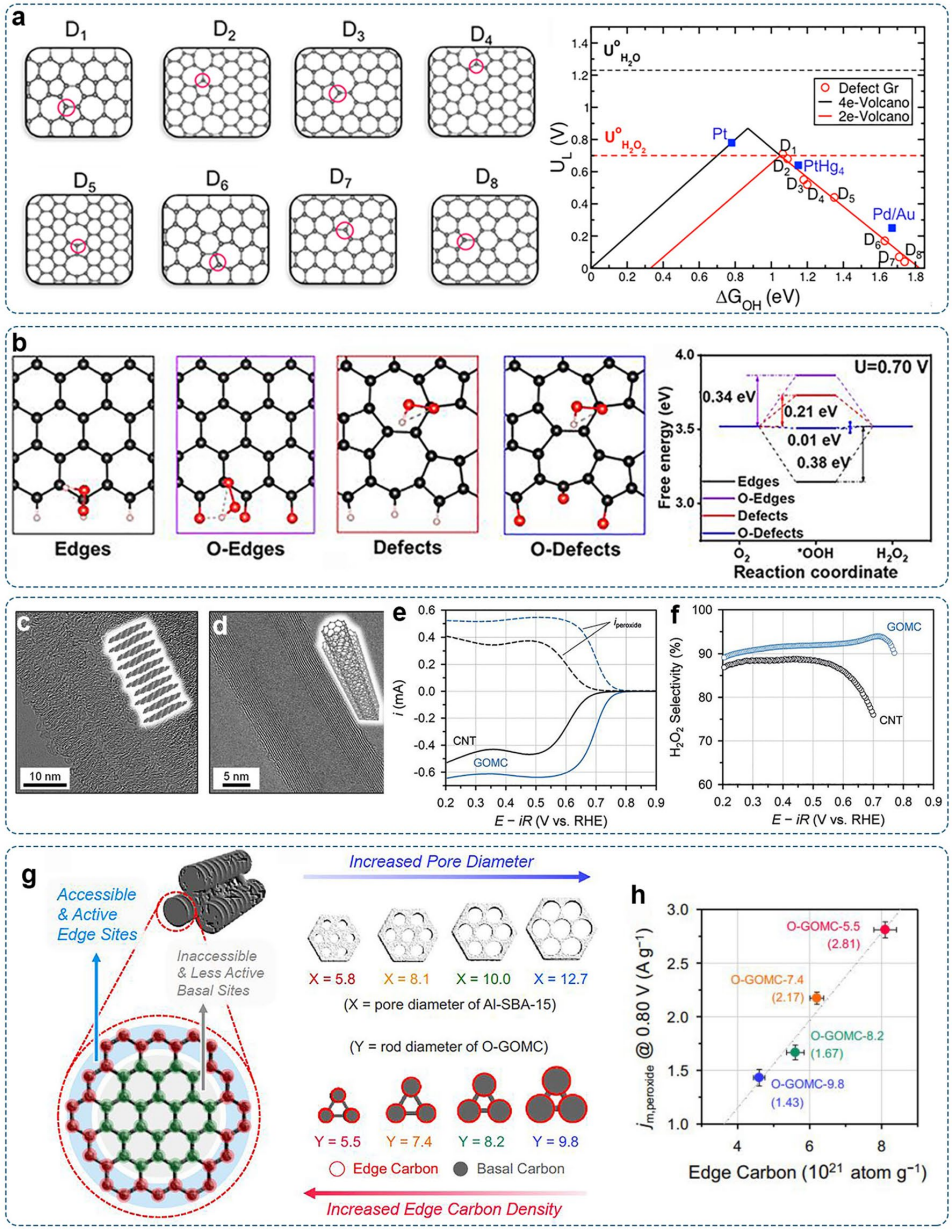
Defect carbon: **a** Different defect configurations examined by DFT calculations and corresponding ORR volcano plots for the 2e^–^ (red line) and 4e^–^ (black line) pathways. Reproduced with permission [134]. Copyright 2018, American Chemical Society. **b** Optimized model structures with the adsorption of *OOH and corresponding free energy profile of 2e^–^-ORR at 0.70 V [138]. Copyright 2021, American Chemical Society. High-resolution TEM image of **c** GOMC and **d** CNT. **e** LSV curves with ring and disk currents of GOMC and CNT in 0.1 M KOH. **f** The corresponding H_2_O_2_ selectivity as a function of applied potentials. Reproduced with permission [139]. Copyright 2022, Wiley–VCH. **g** Illustration of the structural properties of the O-GOMC-Y catalysts. **h** Correlation between the mass activity for H_2_O_2_ production and the edge density in the O-GOMC-Y catalysts. Reproduced with permission [140]. Copyright 2021, Elsevier

There are a variety of structural defect configurations, including holes, edges (e.g., armchair and zigzag edges), vacancies, and a series of topological defects (e.g., pentagons, heptagons, and octagons) [136, 137]. Probing the role of defects on nanocarbon materials’ catalytic performance is essential to understanding the authentic activity origins and providing guidance for further material optimization. Bao and co-workers systemically investigated the effect of different defects on oxygen reduction through DFT calculations [134]. The 2e^–^ (red line) and 4e^–^ (black line) ORR volcano plots were established with the calculated limiting potential as a function of ΔG_*OH_ (Fig. 8a). Several *sp*^*2*^-type defect configurations, especially these double-vacancy defective types with non-hexagonal ring members, exhibited high activity for the 2e^–^-ORR. Wang and co-workers reported the synthesis of defective carbon black (CB) via a one-step plasma method, which could rapidly introduce a high concentration of defects [138]. The resultant CB-A catalyst displayed a selectivity of almost 100% for the electrochemical generation of H_2_O_2_. For theoretical investigations, they proposed that defects with carbonyl function group terminated 555–777 rings (5 and 7 refer to pentagon and heptagon, respectively) exhibited a small theoretical overpotential for H_2_O_2_ (Fig. 8b).

The edge sites of nanocarbon materials have attracted significant attention due to their importance in affecting surface reactivity [141]. Because of edge-induced charge density redistribution, carbon nanostructures with higher edge exposure are more reactive than the basal plane carbons [111]. For example, the edge-rich graphitic ordered mesoporous carbon (GOMC) exhibited ~ 22 times higher mass activity than basal plane-rich carbon nanotubes (Fig. 8c–f) [139]. It could stably produce H_2_O_2_ for 16 h with Faradaic efficiency of 99% and accumulated H_2_O_2_ concentration of 24 ± 2 mM. Moreover, edge sites are favorable for immobilizing active species. The creation and utilization of reactive regions can be maximized by enriching graphitic carbon edges. In the recent study by Joo et al., they used pore-size-tuned Al-SBA-15 templates and synthesized a series of carboxyl and carbonyl group-modified GOMC catalysts (O-GOMC-Y) with the adjustable density of edge carbon sites (Fig. 8g) [140]. The increase in edge density was observed along with the decrease in rod diameter in precursor templates. A linear relationship existed between the edge carbon density and H_2_O_2_ electro-synthesis activity (Fig. 8h). The optimal performance was achieved on the O-GOMC-5.5 sample with the highest edge density and abundant active edge carboxyl and carbonyl groups.

From the above discussion, one could find that the types, locations, configurations, and density of dopants, functional groups, and defects in carbon structure are essential parameters that affect catalytic performance. Although theoretical calculations can help identify the correlations between the structural property and electrocatalytic activity, the detailed mechanisms of carbon-based catalysts still remain elusive. The structural features of carbon materials are critically dependent on the preparation conditions (precursors, pyrolysis temperatures, and duration). However, it is challenging to preciously synthesize carbon nanomaterials with fixed structural features that match well with the theoretical models. Thus, current studies focused on revealing the specific effects of pore structures, defects, doped heteroatoms, and oxygen functional groups cannot solely exclude the contribution from other factors. In fact, synergetic effects resulting from the rational structure design and active site regulation are generally responsible for the observed high activity and selectivity for 2e^–^-ORR on carbon electrocatalysts. More advanced experimental techniques are needed to controllably fabricate model catalysts and reveal the exact activity origin from which kinds of defects/dopants/functional groups and corresponding distribution. In this way, a well-defined structure–property correlation could be built, thus revealing key factors and principles for designing high-performance electrocatalysts.

#### Engineering Porosity of Carbon Catalysts

Porous carbon materials with various morphology and pore size distribution have shown great promise for gas-related electrocatalysis because of their large surface area and high pore/volume ratio, which facilitate the exposure of active sites and mass transport for reactants and products [37, 142]. In the RRDE tests, the generated H_2_O_2_ within the catalysts can rapidly diffuse out via mechanical rotation. The influence of mass transport is not obvious [143]. However, in practical applications such as the flow cell with the catalyst-loaded gas diffusion layer (GDL) electrodes, the residence time of the produced H_2_O_2_ on the catalyst surface would be much longer than in the case of RRDE. Consequently, the produced H_2_O_2_ could be consecutively decomposed on the catalyst [52].

Micropores (< 2 nm) in carbon materials can host active sites and contribute to catalytic activity [145]. The H_2_O_2_ generated at the active sites may be stored within micropores for a longer time due to the small voids. This poses a challenge for the catalysts with rich micropores towards the H_2_O_2_ evolution because it is more likely that the generated H_2_O_2_ goes through further electrochemical reduction or chemical disproportionation, thus reducing the catalysis efficiency [103]. Moreover, when the carbon catalysts are mixed with binder reagent (e.g., Nafion, polytetrafluoroethylene (PTFE)) to fabricate the electrode, some active sites in the micropores might be blocked and inaccessible for electrolytes and reactants. Consequently, active sites exposed to the triple-phase interface are constrained, severely limiting catalytic performance.

**Fig. 9:**
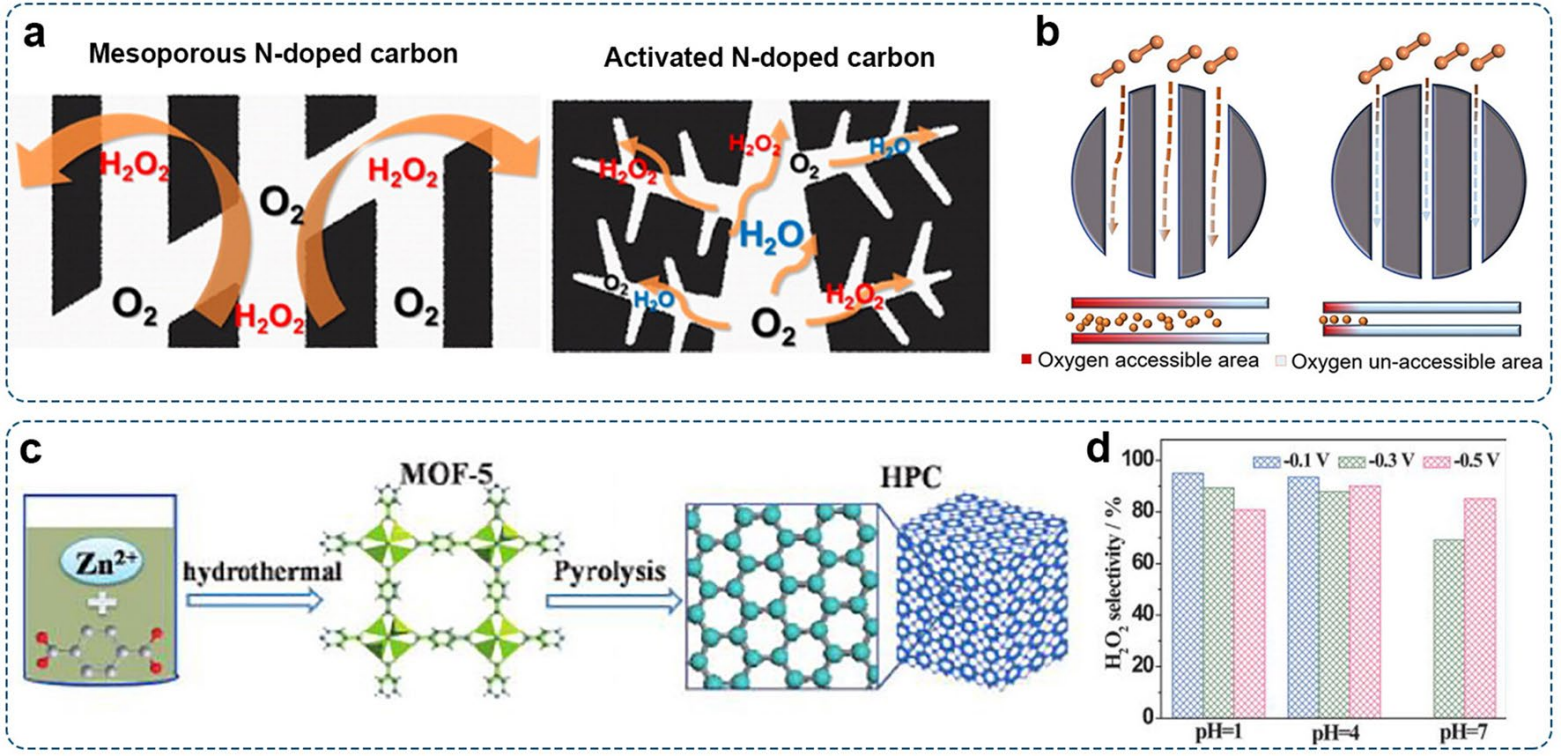
Porous carbon catalysts for H_2_O_2_ production: **a** Schematic illustration of ORR mechanism on mesoporous carbon and microporous carbon. Reproduced with permission [146]. Copyright 2014, American Chemical Society. **b** Schematic illustration of mesopores for facilitating O_2_ transport. Reproduced with permission [144]. Copyright 2021, American Chemical Society. **c** Schematic illustration of HPC preparation. **d** H_2_O_2_ selectivity of HPC-H24 at different pH values. Reproduced with permission [147]. Copyright 2015, Wiley–VCH

In comparison, mesopores (2–50 nm) in carbon materials enable the effective transport of reactants and products toward/away from catalytic sites [145]. With larger pore sizes, H_2_O_2_ can move more rapidly out from the catalyst layer, reducing its residency time and the likelihood of further reduction [134]. Park et al. investigated the correlation between H_2_O_2_ production activity and pore sizes. Micropore-dominant and mesopore-dominant nitrogen-doped carbon materials were fabricated as model catalysts. The mesopore-dominant catalyst showed higher 2e^–^-ORR selectivity than the micropore-rich counterpart (Fig. 9a) [146]. Recently, Huang et al. prepared carbon nanospheres with different pore sizes using phenolic resin spheres as the precursor for studying their ORR performances [144]. The results showed that catalysts with rich mesopores enabled more accessible contact areas for O_2_ and electrolyte ions, thus presenting better 2e^–^-ORR performance (Fig. 9b). In these cases, the beneficial role of mesoporous architecture in carbon materials for sustainable electro-synthesis of H_2_O_2_ has been verified.

However, Čolić et al. investigated the 2e^–^-ORR activity of various commercial porous carbon materials with different porous structures [148]. They observed that carbon materials with a higher amount of micropores displayed higher H_2_O_2_ productivity, while activity for the 4e^–^-process was not affected by the micropore level. In another study by Iglesias et al., reduced 2e^–^-ORR selectivity was observed for N-doped graphitized carbon nanohorns when the microporous volume decreased [114]. They claimed that the O_2_ molecules trapped in micropores with high concentrations would occupy the active sites, thus decreasing the availability of active sites for further reduction of H_2_O_2_. Controversy may arise from other possible factors linked with the micro-society of carbon structures. For example, carbons with higher amounts of micropores may exhibit more defects and edge sites, which are inseparable from carbon materials and have shown an influence on ORR activity.

Hierarchically porous carbon (HPC) materials comprised of micro-, meso-, and macropores can achieve a good balance between activity/selectivity and mass transfer. As reported by Chen et al., hierarchical porous carbon (HPC) derived from the carbonization of metal–organic framework (MOF) was used to selectively reduce O_2_ to H_2_O_2_ (Fig. 9c) [147]. The obtained HPC-H24 sample possessed a high surface area of 2,130 m^2^ g^−1^ with interacted micropores, mesopores, and macropores. Micropores could offer abundant catalytic sites (e.g., carbon defects), whereas mesopores and macropores could minimize the diffusion resistance for mass transport. As a result, The H_2_O_2_ selectivity of HPC-H24 is 80.9–95.0% in acidic solution (pH = 1 and 4) and 70.2–85.1% in neutral solution (Fig. 9d).

Despite these, further efforts should provide deep insights into how the porous structure (e.g., pore size, volume, distribution, and connectivity) affects the ORR performance beyond the aspects mentioned above, thus establishing the general design principle of porous carbon materials for targeted electrocatalysis reactions.

### Carbon-Supported Single-Atom Catalysts

With the prominent advantages of maximum atom-utilization efficiency, homogeneous reactive sites, and encouraging activity, nonprecious transition-metal single-atom catalysts (SACs) have received considerable attention and have been widely applied in heterogeneous catalysis [54]. The atomically dispersed metal sites are usually anchored by or coordinated with the surface atoms of host materials [54]. Particularly, carbon-based materials (such as graphene, porous carbon, and carbon nanotubes) serve as ideal and accessible substrates to immobilize atomic metal species. Their graphitic frameworks with a high surface area can prevent metal agglomeration and provide the catalyst with outstanding stability and excellent conductivity [149]. Such merits are responsible for the enhancement of electrocatalytic performances. However, the pristine carbon materials often show weak metal-support interactions. Thus, surface modifications such as introducing defects and heteroatoms (e.g., N, S, O) are extensively applied to enhance the interaction between metal atoms and carbon substrates and thus stabilize the atomic metal sites. The representative example is metal–nitrogen–carbon (M–N–C, M = Fe, Co, Ni, etc.) materials. The atomic metal atoms coordinate with surrounding N atoms, forming well-defined M–N_*x*_ moieties [150]. A typical coordination structure is the M–N_4_ moiety with typical four-coordinated metal phthalocyanine- or porphyrin-like configuration, which is responsible for the high catalytic activity as widely demonstrated by theoretical and experimental studies [151].

#### Adjusting the Central Metal Atoms

**Fig. 10:**
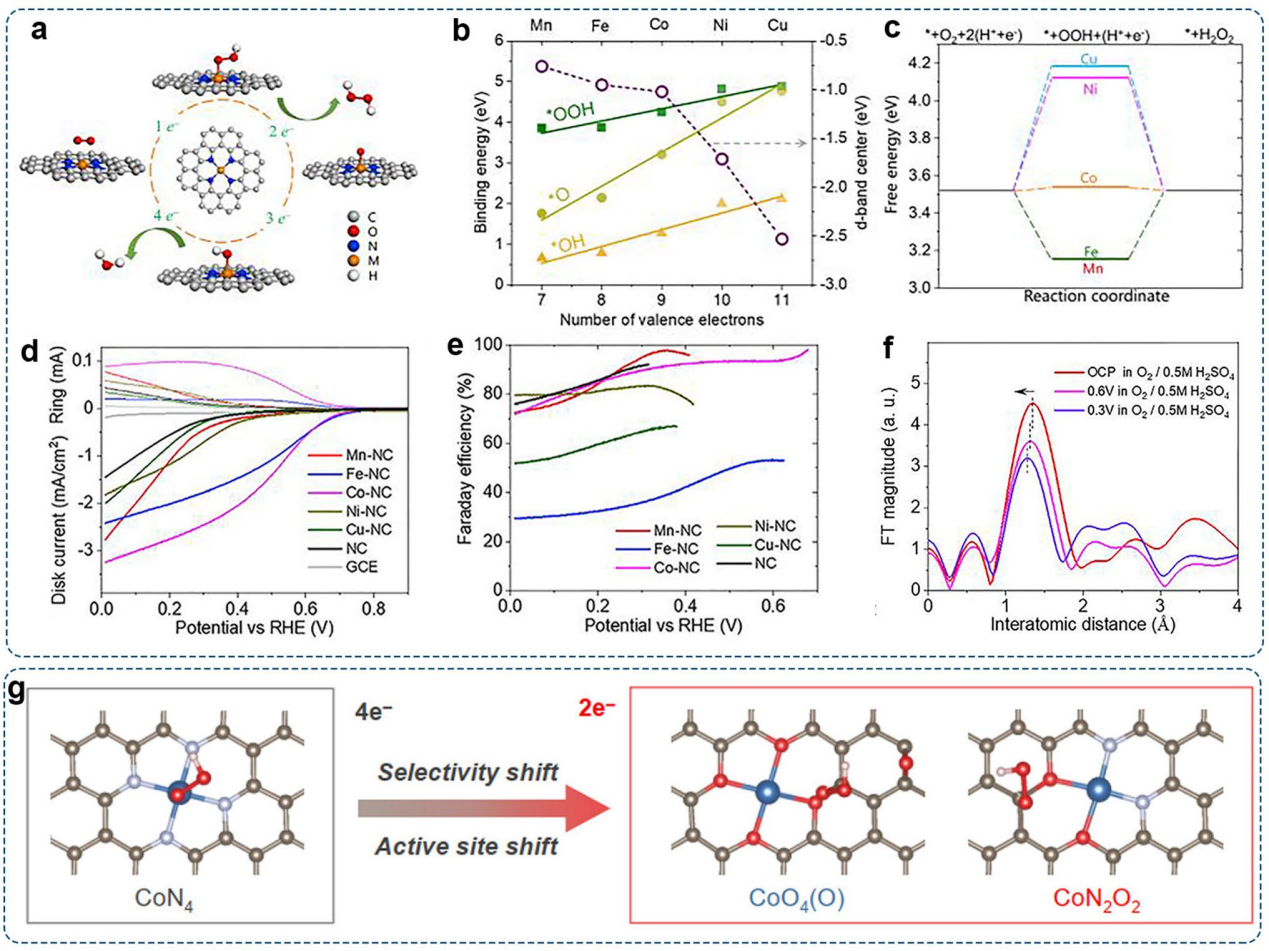
Investigation of Co–N–C SACs for the 2e^–^-ORR: **a** Schematic illustration of ORR pathway on M–N–C SACs (M = Mn, Fe, Co, Ni, and Cu). **b** Binding energy of *OOH, *O, and *OH on M–N–C SACs and *d*-band center of M atom. **c** Free energy diagrams of 2e^–^-ORR on the M–N–C SACs. **d** LSV curves of different M–N–C catalysts in O_2_-saturated 0.1 M HClO_4_. **e** Selectivity for 2e^–^-pathway as a function of applied potential. **f** Operando EXAFS spectra of Co–NC collected during ORR operation. Reproduced with permission [33]. Copyright 2020, Elsevier. **g** The shift of active site and ORR pathway on CoN_4_, CoO_4_(O), and CoN_2_O_2_ moieties. Reproduced with permission [156]. Copyright 2021, American Chemical Society

In most cases, the isolated metal atoms directly interact with oxygen-containing intermediates during the ORR [24]. Thus, the catalytic properties of M–N–C SACs highly depend on the central metals. Changing the central metal is a simple and direct method to tailor the activity and selectivity of SACs.[152]. By combining theoretical and experimental methods, Gao et al. systematically studied the relationship between the structure of atomic Mn, Fe, Co, Ni, and Cu anchored nitrogen-doped graphene and their 2e^–^-ORR performances (Fig. 10a) [33]. DFT calculations revealed that the binding energies of *OOH, *O, and *OH were generally scaled with the number of valence electrons in the M atom (Fig. 10b–c). The *d*-band centers of the M atoms presented a down-shift from Mn to Cu, leading to weaker binding of reaction intermediates. The experimental results showed that Co–NC catalyst with the optimized-band center behaved as a highly active and selective 2e^–^-ORR electrocatalyst with high Faraday efficiency > 90% (Fig. 10d–e). Further operando X-ray absorption spectroscopy (XAS) was employed to track the dynamic change of the Co–N–C active center under reaction conditions. The result showed that the Co–N bonding distance experienced elastic compression with the shortened 0.03 Å from 0.6 to 0.3 V (vs. RHE) and then bounded back to its initial value (1.35 Å) when the potential returned to the open-circuit condition. Thus, the nitrogen-coordinated single Co atom was recognized as the reactive center (Fig. 10f). Similarly, other groups also observed the 2e^–^-pathway over Co–N–C SACs [153–155].

#### Engineering the Coordination Environment

However, Co–N–C SACs have also demonstrated highly efficient 4e^–^-ORR activity [42, 157]. Such a controversy can be regarded as a lack of a comprehensive understanding of catalyst structures and reaction mechanisms. In M–N–C SACs, the geometric structures of catalysts, including the coordination number, the N-doping configurations (pyridinic N vs. pyrrolic N), locations of the metal site (edge vs. basal plane), binding pockets of the second coordination sphere, and guest groups on the host materials, are essential parameters that can affect the local electronic environment of active center, and eventually influence the intrinsic activity and corresponding reaction pathway [158]. For example, Shao et al. found that Co–N_5_ coordination configuration with an extra vertical Co–N bond was more favorable for the 2e^–^-ORR than conventional Co–N_4_ sites in Co–N–C SACs [159]. Liu’s group reported that the isolated Co atom coordinated with four pyrrolic N atoms was mainly responsible for the 2e^–^-ORR, while the pyridine-type Co–N_4_ site preferred the 4e^–^-ORR [160]. Recently, our group investigated the edge effect of the carbon substrate on regulating the ORR pathway over the Co–N–C SACs [161]. Atomic Co–N_4_ sites over the edges of hierarchically porous carbon were more selective for the 2e^–^-ORR than basal-plane Co–N_4_ sites anchored on graphene flakes.

Nevertheless, precisely regulating the configurations and microenvironment of active sites is hard to guarantee [162]. The local structures of Co–N–C SACs derived from various precursors (e.g., C, N, and Co sources) and synthetic methods (e.g., pyrolysis temperature and duration) may be significantly different from case to case, resulting in conflicting results in catalytic performances (2e^–^ or 4e^–^). Thus, the geometric structure of SACs should be carefully characterized to identify the actual active configuration.

It is evident that the surrounding coordination environments play a significant role in modulating the electronic structure and catalytic behavior of the active site [163]. With the variation of coordinative dopants in carbon supports, other elements besides N (e.g., O, B, S, P) have been doped into the carbon framework, further tailoring electronic states of the metal center and manipulating the reaction pathway for targeted products. Qiao et al. fabricated a new kind of Mo SACs (Mo_1_/OSG-H), which possessed a distinctive Mo metal center with O and S dual coordination [164]. The electrochemical tests showed that the H_2_O_2_ selectivity of Mo_1_/OSG-H was over 95% in 0.10 M KOH. The corresponding electron transfer number was calculated to be 2.1 for a wide range of applied potentials, revealing a highly selective 2e^–^-pathway. Zhang et al. reported a metal-Schiff base-like electrocatalyst (Ni-N_2_O_2_/C) containing atomic Ni centers coordinated by two O atoms and two N atoms, as evidenced by XANES.[165]. Compared with Ni-N_4_/C, the selectivity of H_2_O_2_ production over Ni-N_2_O_2_/C reached a maximum of ~ 96%. These works verified the crucial role of the local coordination structure in SACs for targeted electrocatalytic performance.

It should be noted that binding sites for reaction intermediates are not always atomic metal moieties. For instance, Qiao et al. reported a Co-SAC with N, O-dual coordination (CoNOC), which demonstrated outstanding activity and selectivity of > 95% [156]. The DFT calculations showed that the optimized *OOH adsorption site on the CoN_2_O_2_ structure was the C atom adjacent to the coordinated O atom. When SCN^−^ ions were added into O_2_-saturated 0.10 M HClO_4_, the ORR activity of CoNOC was almost unchanged. By contrast, significant activity degradation was observed for the CoNC catalyst with Co–N_4_ moiety. These results indicated that the O-adjacent C atom in CoNOC was responsible for the 2e^–^-ORR pathway rather than the Co atom (Fig. 10g). Therefore, the reactive center of catalysts should be carefully examined via comprehensive characterizations.

#### Altering Environmental Atoms

Environmental atoms on the second or even higher coordination shells are not directly bonded to the central metal atoms. However, they can influence the electronic structure of active sites through long-range delocalization. For example, Hyeon et al. modified Co–N_4_ moiety with electron-rich epoxy groups on nitrogen-doped graphene for H_2_O_2_ production (Co_1_-NG(O)) [166]. As revealed by DFT calculations, the charge state of the Co atom became more positive when O atoms were connected by two C atoms near the Co–N_4_ moiety (Co–N_4_(O) and Co–N_4_(2O)). The ∆G_*OOH_ value was increased, facilitating the 2e^–^-ORR pathway. The electrochemical tests showed that the synthesized Co_1_-NG(O) catalyst displayed enhanced activity for H_2_O_2_ production. Particularly, a high kinetic current density of 2.8 mA cm^−2^ and mass activity of 155 A g^−1^ were achieved at 0.65 V (vs. RHE) with negligible activity loss over 110 h. They used Co_1_-NG(O) in the electrochemical H_2_O_2_ production device, enabling high H_2_O_2_ productivity of 418 ± 19 mmol g_cat_^−1^ h^−1^.

**Fig. 11:**
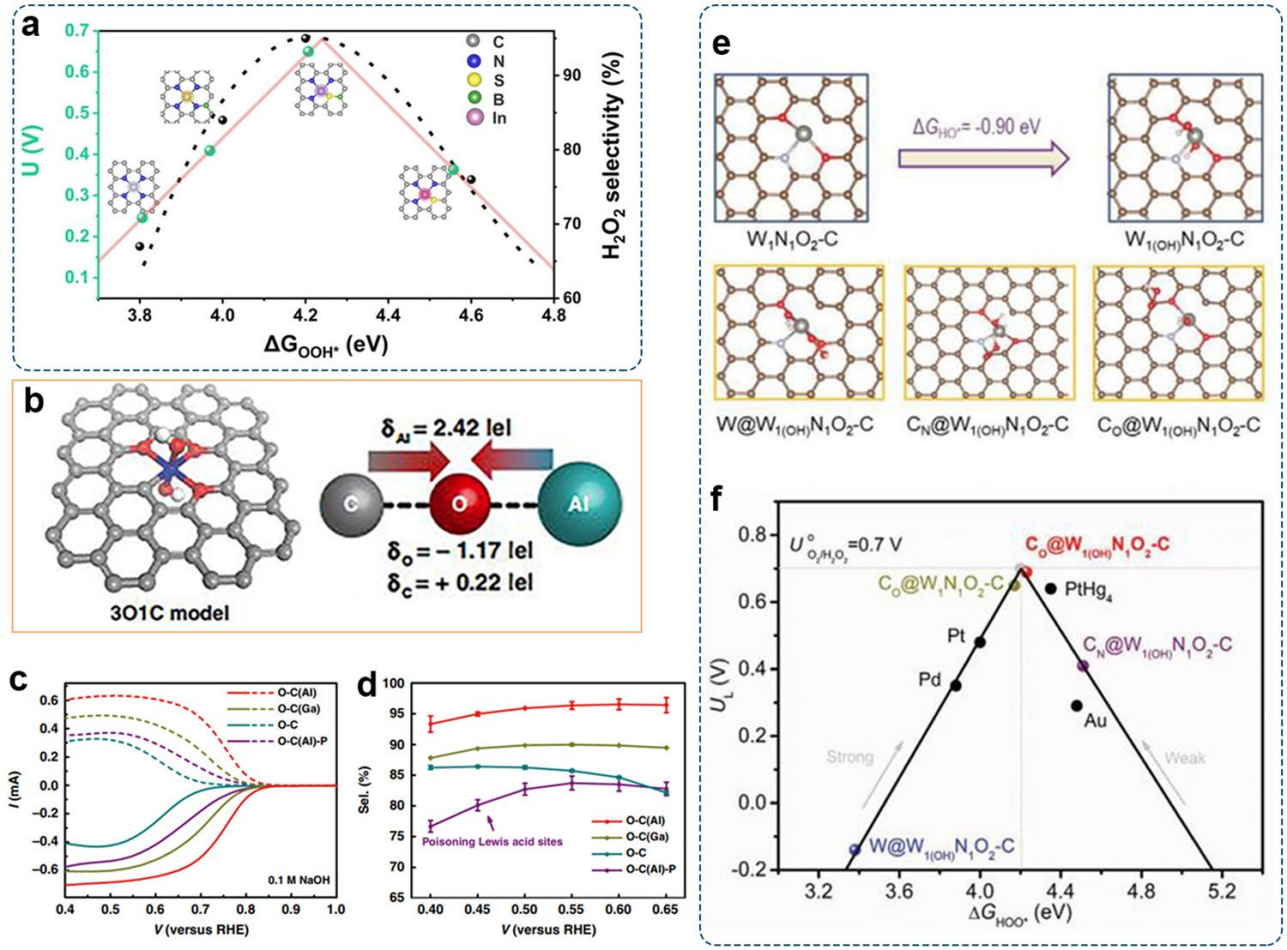
The influence of environmental atoms and guest groups on H_2_O_2_ selectivity: **a** Activity volcano plot of different atomic configurations toward H_2_O_2_ production. Reproduced with permission [168]. Copyright 2021, Wiley–VCH. **b** Proposed structure model with the 3O1C configuration and charge transfer between Al and adjacent carbon atom. Blue, red, white, and gray balls denote metal, O, H, and C atoms, respectively. **c** LSV curves with ring and disk currents of O-C(Al), O-C(Cr), and O-C(Ga) in 0.1 M NaOH. **d** The corresponding H_2_O_2_ selectivity as a function of the applied potential. Reproduced with permission [169]. Copyright 2020, Springer Nature. **e** Schematic of the adsorption of *OH on the W atom of W_1_N_1_O_2_-C coordination configuration and atomic configurations of *OOH adsorption on different sites. **f** The calculated activity volcano plot for the 2e^–^-ORR. Reproduced with permission [170]. Copyright 2021, Wiley–VCH

Compared with d-block transition metals, the main group metals were thought to be less active in catalytic reactions due to the fully filled *d*-orbitals. Recently, it has been found that s/p electrons in the main group (s- and p-block) metals could also be tuned via engineering the modification of environmental atoms, making them promising in catalytic reactions [167]. Via the combination of DFT simulation and experimental characterizations, Li’s group reported In single-atom moieties on the hollow carbon rods (In SAs/NSBC) as a 2e^–^-ORR catalyst [168]. The first-coordinated N, S, and second-coordinated B resulted in the optimized electronic structure of the InN_3_SB center with suitable adsorption energy for the key *OOH intermediate (Fig. 11a). Accelerated reaction kinetics and high selectivity (> 95%) were achieved in alkaline and neutral media.

#### Introducing Guest Groups

The additionally introduced guest groups (e.g., functional groups, small molecules, or ligand anions) on the host materials may also influence the catalytic properties through the coordinate bonds or intermolecular interactions [156, 166, 171]. For instance, SACs with both Co–N–C sites and oxygen functional groups have been reported to exhibit excellent catalytic performance for H_2_O_2_ production with high activity and selectivity [161, 172].

Meanwhile, the tight binding of intrinsic intermediate on the metal center would serve as internal guest groups and result in the self-adjusting of active sites, thus influencing the electrocatalysis process [173, 174]. Chen et al. fabricated the group IIIA metal (Al, Ga, Cr)-doped oxidized carbon materials with Lewis acid site (octahedral M–O motifs) via the pyrolysis of metal–organic framework MIL-53(Al, Ga, Cr) [169]. In this work, the metal sites (e.g., Al and Ga) were thought to be catalytically inactive due to the strong binding of the *OH. The carbon atom adjacent to metal was suspected to be the active site toward 2e^–^-ORR due to the charge transfer between the oxygen-doped carbon layer and metal moiety (Fig. 11b). They found that the selectivity for 2e^–^-ORR over O–C(M) demonstrated a positive linear correlation with its Lewis acidity (O–C(Ga) < O–C(Cr) < O–C(Al)). The optimal O–C(Al) catalyst with the stronger Lewis acidity exhibits superior activity and selectivity (> 95%) for the 2e^–^-ORR in alkaline media (Fig. 11c–d). Wang et al. immobilized 5d transition metal W atoms on the O, N-doped carbon matrix (W_1_/NO-C) [170]. Via careful characterizations with aberration-corrected scanning transmission electron microscopy (AC-STEM), XAS, and XPS analyses, an unusual terdentate W_1_N_1_O_2_ moiety with coordination of two O atoms and one N atom was identified. W_1_/NO-C catalyst with terdentate W_1_N_1_O_2_-C coordination structure exhibited high catalytic efficiency for the 2e^–^-ORR, enabling the production rate of ~ 1.23 mol g_cat_^–1^ h^–1^ and Faradaic efficiency of ~ 5%. Additionally, DFT calculations revealed that OH^–^ could be attached to the W center in alkaline media due to the negative ΔG_*OH_ of –0.9 eV (Fig. 11e). Further screening found that the O-adjacent C atoms in the *OH modified W_1_N_1_O_2_-C moiety were more active toward the 2e^–^-ORR (Fig. 11f). Evoked by these works, the self-adjusting effects induced by intrinc ORR intermeiates should be exploited as a new design route for regulating SACs toward specific reaction pathways.

Given the diverse choices of metal centers, support materials, and non-metal dopants, the categories of SACs will be further extended. The established approaches, including changing the transition metal center, adjusting the local coordination environment, altering environmental atoms, and introducing guest groups, can tailor the active site with a suitable binding affinity toward oxygen intermediates [175, 176]. The flexible tunability of SACs provides a limitless opportunity to achieve high selectivity and productivity for H_2_O_2_ production simultaneously.

Besides the catalytic activity, the chemical stability of catalysts has a profound impact on long-term and industrial H_2_O_2_ production. A recent study suggests that the surface oxidation and hydroxylation of SACs caused by long-term storage could affect the ORR activity [177]. Besides, the corrosion of carbon matrix arose from H_2_O_2_ or other radical chemical attacks, and corresponding determinantal effects have been recognized in other ORR techniques, such as fuel cells and Li-O_2_ batteries. As discussed in Sect. 3.3.2, proper oxidation of the carbon framework would benefit H_2_O_2_ production. Nonetheless, chemical changes of carbon substrates during the ORR operation and corresponding influences on the 2e^–^-ORR performance of SACs have not yet been identified and undoubtedly require future efforts. Previous efforts to characterize SACs have mainly focused on the local coordination environment of the metal sites [158]. Recent studies confirm that reactant/intermediate adsorption and applied potential may drive the dynamic evolution of the M–N_*x*_ moieties [178]. Thus, structural changes induced during the electrocatalytic process should receive considerable attention in future studies.

### Molecular Electrocatalysts

#### Metal-Containing Molecular Electrocatalysts

For decades, various organometallic complexes have been developed as catalysts for diverse electrocatalytic reactions in both aqueous and non-aqueous electrolytes [179, 180]. As these molecular metal complexes usually consist of atomic metal centers linked by organic ligands with well-defined coordination environments, they have also been regarded as the early example of SACs [149].

Previous studies suggest that redox properties are responsible for ORR, and the lower redox potential of metal complexes enables higher H_2_O_2_ selectivity [181]. The redox properties of these compounds strongly depend on the electronic structures of the metal center and corresponding metal–ligand interactions [182]. This means that the electrocatalytic properties of molecular metal complexes can also be finely tailored by changing the metal centers and ligands. For example, the ORR catalyzed by Fe-based molecular complexes commonly proceeds via the 4e^–^-process, whereas the 2e^–^-process has been observed on Co-based molecular complexes (e.g., Co porphyrin). Moreover, the presence of substituent functional groups on the inner rings of macrocycle compounds can affect these materials’ electrochemical and chemical properties. One possible explanation is that electron-withdrawing/donating substituents could alter the electron densities of the metal-ion centers, hence changing the redox properties [183]. Therefore, via the careful regulation of metal centers and ligands, a wide range of molecular complexes with Fe [184, 185], Ni [186],Cu[187], and Mn [188, 189]. centers have also been observed with 2e^–^ ORR activity.

**Fig. 12:**
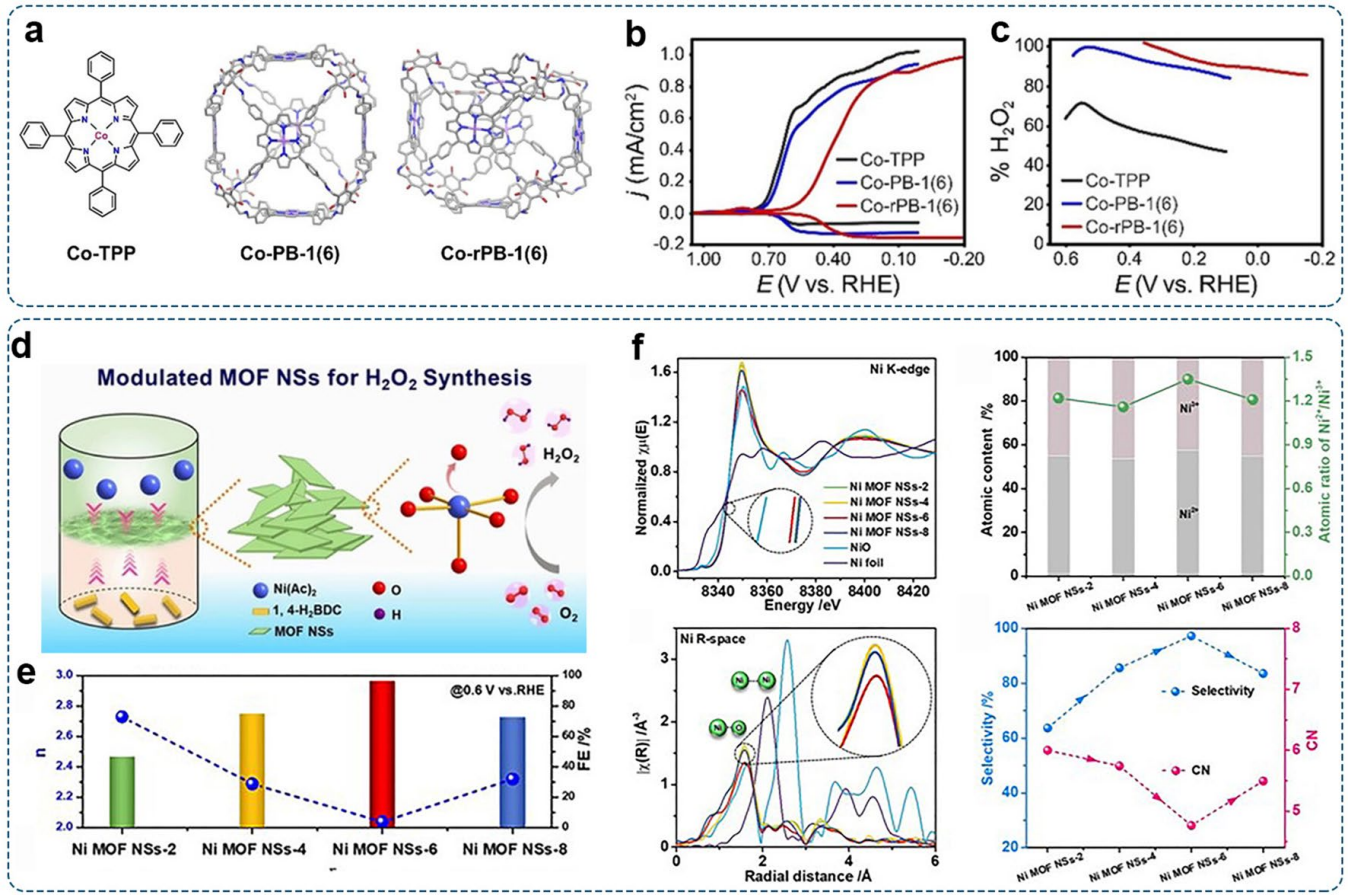
Investigation of metal-containing molecular electrocatalysts: **a** Chemical structure of Co-TPP, Co-PB-1(6), and Co-rPB-1(6). **b** LSV curves with ring and disk currents of Co-TPP, Co-PB-1(6), and Co-rPB-1(6) in phosphate buffer solution (pH = 7). **c** The corresponding H_2_O_2_ selectivity as a function of applied potentials. Reproduced with permission [191]. Copyright 2020, Wiley–VCH. **d** Schematic illustration of the Ni MOF NSs with partially unsaturated Ni sites for H_2_O_2_ synthesis. **e** The number of transferred electrons and the selectivity obtained at 0.6 V (vs. RHE). **f** Ni K-edge XANES and FT-EXAFS analyses for different Ni MOF NSs and reference samples. Reproduced with permission [192]. Copyright 2021, Wiley–VCH

Geometric effects, such as distance and angle between the metal centers, are important in determining the activity and selectivity. It has been reported that Co-cofacial complexes linked by ligands in a co-facial configuration could bind both dioxygen atoms via Co–O–O–Co and induce the 4e^–^-ORR process [190]. In contrast, monomeric Co complexes presented higher selectivity for 2e^–^-ORR due to the presence of isolated active sites [28]. Thus, to enhance the 2e^–^-selectivity, the aggregation of monomer that creates intermolecular active sites should be avoided. Chang et al. developed a supramolecular strategy for promoting the selective reduction of O_2_ for the electrosynthesis of H_2_O_2_ [191]. Cobalt tetraphenylporphyrin (Co-TPP) was utilized as a building block to assemble the permanently porous supramolecular cage (Fig. 12a). The resultant porphyrin boxes, Co-PB-1(6) and Co-rPB-1(6) could achieve 90%–100% selectivity in the neutral pH media (Fig. 12b–c). They attributed this high H_2_O_2_ selectivity to site isolation of the discrete molecular units in each supramolecule.

Conductive metal–organic frameworks (MOFs), assembled from metal ions and organic linkers with periodical arrangements, are attractive candidates in the 2e^–^-ORR. (Ni_3_(HITP)_2_ (HITP = 2, 3, 6, 7, 10, 11-hexaiminotriphenylene) with square planar Ni–N_4_ sites was reported for 2e^–^ ORR and presented the H_2_O_2_ selectivity of 63%–75% in the potential range from 0.55 to 0.75 V (vs. RHE) [193]. Huang et al. fabricated 2D Ni MOF nanosheets (NSs) with controlled molar ratios of metal precursors and organic linkers via a mild liquid–liquid interfacial reaction method (Fig. 12d) [192]. When directly used as electrocatalysts for the 2e^–^-ORR in alkaline electrolyte, the partially unsaturated Ni MOF NSs-6 catalyst displayed an outstanding 2e^–^ selectivity of 98% (Fig. 12e). As revealed by XAFS, the catalytic activity was correlated with the number of unsaturated coordinated metal sites in Ni MOFs (Fig. 12f).

#### Metal-Free Organic Molecular

**Fig. 13:**
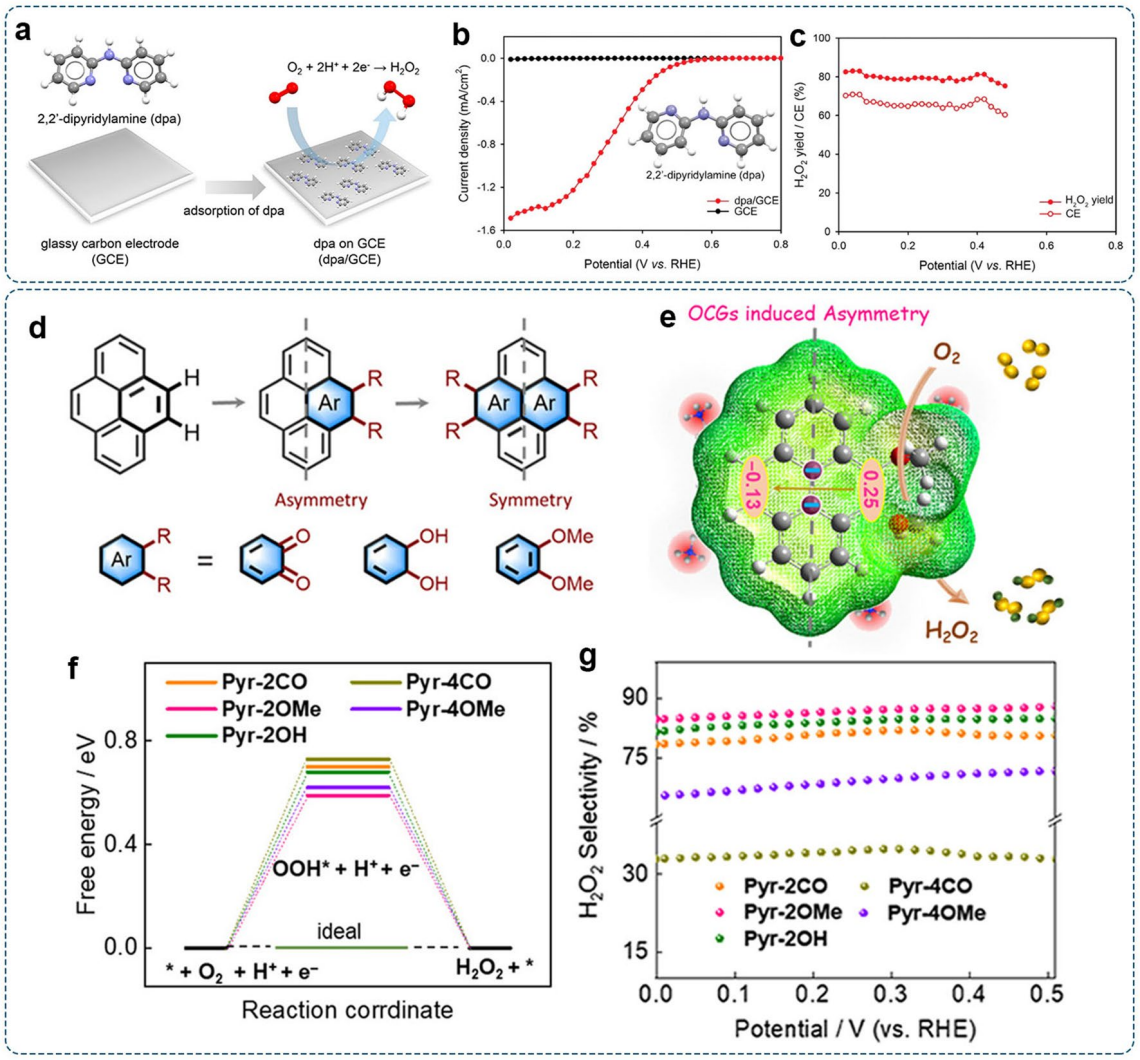
Investigation of metal-free molecular electrocatalysts: **a** Schematic illustration of 2,2′-dipyridylamine (dpa) for the 2e^–^-ORR. **b** LSV curves recorded on RRDE and **c** corresponding H_2_O_2_ selectivity. Reproduced with permission [196]. Copyright 2019, American Chemical Society. **d** Molecular structures of pyrene with different functional groups. **e** Schematic illustration of Pyr-2OMe for the 2e^–^-ORR. **f** The free energy of pyrene-based molecules for the 2e^–^-ORR. **g** The H_2_O_2_ selectivity of pyrene-based molecules as a function of applied potentials. Reproduced with permission [200]. Copyright 2019, Elsevier

Though active toward the 2e^–^-ORR, no complete consensus on the stability of metal-containing molecular catalysts has been reached yet [194]. Apart from the possible detachment of molecular catalysts from electrode surfaces, the dissolution of metallic ions could induce Fenton or Fenton-like reactions, resulting in the decomposition of H_2_O_2_. The generated active oxygen species (e.g., ·OH and ·OOH) would, in turn, destroy organic compounds, thus causing the degradation of the catalytic performance. Some metal-free organic molecules could be a suitable choice to avoid this issue. Wen and co-workers synthesized 2D redox-active cationic covalent triazine network, which could undergo the reversible 2e^–^ redox process to mediate the ORR to form H_2_O_2_ [195]. The H_2_O_2_ selectivity could reach ∼85% in the alkaline electrolyte. Zelenay’s group reported 2,2′-dipyridylamine (dpa) as the ORR catalyst (Fig. 13a). An onset potential of ∼0.60 V (vs. RHE) with the 2e^–^-selectivity of ∼80% was achieved in the acidic aqueous electrolyte (Fig. 13b–c) [196]. Warczak et al. reported N,N’-dimethyl perylenetetracarboxylic diimide (PTCDI) as an organic semiconductor catalyst for electrochemical generation of H_2_O_2_ in a pH range of 1–13, with a production rate up to 26 kg h^–1^ H_2_O_2_ per gram catalyst [197]. Conductive polymers with mixed metal and polymer-like properties are considered in electrocatalytic applications due to their high electronic conductivity and distinct redox properties [198]. Poly(3,4-ethylenedioxythiophene) (PEDOT) electrode, reported by Mitraka et al., enabled the continuous generation of high concentrations of peroxide with Faraday efficiency remaining close to 100% [199]. The activity of the metal-free molecular catalyst is also highly dependent on its molecular structure. Recently, Yang et al. modified pyrene with −OH, −C=O, and −OCH_3_ groups (Fig. 13d) [200]. The asymmetric molecular structures would alter the local charge redistribution and large dipole moments (Fig. 13e). As a result, Pyr-2CO, Pyr-2OH, and Pyr-2OMe with asymmetric structures presented better 2e^–^ activity than Pyr-4CO and Pyr-4OMe with symmetrical structures. Remarkably, the electron-donating −OCH_3_ groups endowed its adjacent carbon atom with the lowest free energy barrier for *OOH formation (Fig. 13f). The H_2_O_2_ selectivity of Pyr-2OMe could reach 88% in 0.1 M KOH (Fig. 13g).

Overall, by taking advantage of tailorable molecular structures, molecular catalysts serve as a powerful platform for developing efficient electrocatalysts with high activity and selectivity. The simple and well-defined structures manifest a clear structure–activity relationship, providing a basis for investigating the nature of active sites and reaction mechanisms. It should be mentioned that the stability of molecular electrocatalysts should be further examined. The reductive oxygen species, like OOH^−^ and ·OOH, are the strong nucleophiles [201], which would induce the decomposition of organic compounds via nucleophilic attack or H-abstraction, thus degrading the catalytic performances [202].

## Interfacial Factors and Engineering

In addition to the inherent structural and electronic properties of electrocatalysts, the triple-phase interface among O_2_, catalyst surface, and electrolytes can significantly affect the overall catalytic performance regarding the mass transport and local reaction environment [203]. Interfacial engineering refers to modifying the local reaction environment of existing active sites, including adjusting the surface wetting states and tailoring the solid–liquid interface. These strategies promise the optimization of catalytic activity, selectivity, and durability beyond engineering electronic and geometric structures of catalyst materials.

The aeration-based electrochemical system for H_2_O_2_ production involves complex triple-phase boundaries among O_2_, solid catalysts, and electrolyte solutions. A sufficient and stable triple-phase is an essential factor affecting the mass transfer and the overall performance of electrochemical H_2_O_2_ production. The surface-wetting state of the catalyst that plays a crucial role in determining the nature of the triple-phase interface should be considered. The hydrophobicity of the catalyst layer can maintain the O_2_ diffusion channel for reactant feeding. Since protons in the aqueous electrolyte also participate in the electrocatalytic process, the hydrated environment around the active sites is required [4].

**Fig. 14:**
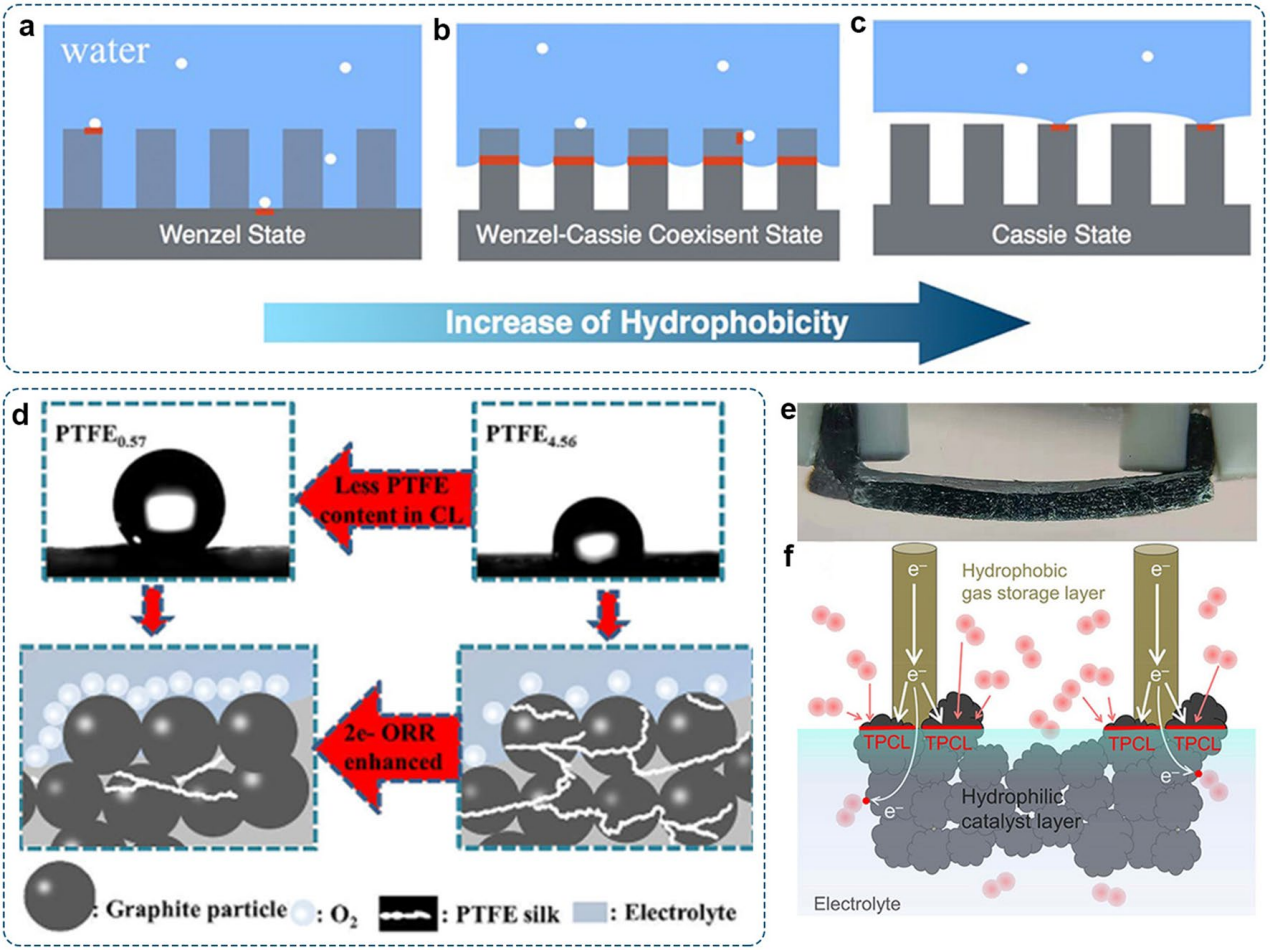
The effects of surface hydrophilicity/hydrophobicity: **a–c** The schematic diagram of three types of surface wetting states. Reproduced with permission [205]. Copyright 2016, Wiley–VCH. **d** Schematic illustration of adjusting hydrophilicity/hydrophobicity by using PTFE to improve the 2e^–^-ORR activity. Reproduced with permission [208]. Copyright 2019, American Chemical Society. **e** Side-view photograph and **f** schematic illustration of the Janus electrode submerged in the electrolyte. Reproduced with permission [209]. Copyright 2020, American Chemical Society

The surface wetting state of catalysts based on the increase of hydrophobicity can be classified into (Fig. 14a–c): (1) Wenzel state: the liquid thoroughly wetted the solid without trapped air at the liquid/solid interface; (2) Wenzel-Cassie coexistent state: the liquid partially intruded into the textured solid surface with a certain amount of trapped air at the interface; (3) Cassie state: the liquid hardly touched the textured surface, giving rise to a quasi-continuous gas layer over the textured solid surface [204, 205]. The Wenzel-Cassie coexistent state has been proposed to facilitate ORR due to its maximal and stable solid–liquid-gas three-phase interface that is favorable for gas diffusion [205]. In RRDE tests, gaseous O_2_ is continuously supplied to saturate the aqueous solution. The aqueous O_2_ (aq), dissolved in the bulk electrolyte, is the oxygen source [206]. Therefore, more electrolyte-catalyst contact arising from the hydrophilic catalyst surface can provide sufficient reactant supply. When practically applied in reactors for large-scale production of H_2_O_2_, O_2_ supply would become the limiting factor due to the limited solubility of O_2_ in the aqueous solutions. The external oxygen-pumping equipment is usually needed to deliver O_2_ sustainably for a higher H_2_O_2_ production rate [207]. In this catalysis system, gaseous O_2_ is supplied directly to the catalyst layer. A hydrophobic electrode surface to maintain the gas diffusion channel benefits O_2_ capture. Li and co-workers investigated the correlation between the hydrophilicity/hydrophobicity of the catalyst layer and the selectivity of the 2e^–^-ORR (Fig. 14d) [208]. PTFE emulsion was dropped on the carbon catalyst layer to adjust the hydrophilicity/hydrophobicity. The optimal PTFE_0.57_ electrode with superhydrophobic feature had the highest H_2_O_2_ yield of 3,005 ± 58 mg L^−1^ h^−1^ at 25 mA cm^−2^ and the highest current efficiency of 84% at 20 mA cm^−2^. In comparison, higher PTFE content reduced the H_2_O_2_ yield and increased the total impedance to hinder electron transfer during the ORR.

To mitigate O_2_ mass transport limitation and enhance O_2_ utilization, Zhang et al. fabricated a Janus electrode with opposite wettability on the adjacent sides [209]. Different from merely hydrophilic or hydrophobic electrodes, the resultant Janus electrode integrated the advantages of sufficient catalyst-electrolyte contact in the hydrophilic catalyst layer and adequate oxygen supply from the hydrophobic gas storage layer. The three-phase contact lines (TPCL) were formed for oxygen transfer instead of relying on the diffusion of dissolved oxygen (Fig. 14e–f). Consequently, an energy-efficient and cost-effective H_2_O_2_ generation was achieved with a production rate of 61.2 ± 0.9 mg L^−1^ at 30 min.

The aerophilic feature of GDL facilitates the trap of O_2_ near the catalyst layer and thus enhances the utilization of O_2_ flow. Recently, Du and co-workers used PTFE to modify the carbon black/carbon felt electrode, resulting in a superhydrophobic natural air diffusion electrode (NADE) to improve the oxygen diffusion coefficient greatly [210]. With the use of NADE, atmospheric air could naturally diffuse into the catalytic layer, resulting in fast H_2_O_2_ production (101.67 mg h^−1^ cm^−2^) with a high oxygen utilization efficiency (44.5%–64.9%) without extra pumping oxygen/air.

## Modulating the Electrolyte Environment

The performance of 2e^–^-ORR electrocatalysts is strongly affected by the electrolyte used. Aqueous solutions can afford abundant proton supply and are typically used for most 2e^–^-ORR studies. Several factors are associated with the local reaction environment of the electrocatalyst immersed in the aqueous electrolyte, including pH, composition, and concentration of cations and anions. Their influences are not always straightforward to map due to intertwining effects, posing challenges to studying electrolyte effects.

The most direct influence of pH is the stability of electrocatalysts. Most transition metal and metal oxide-based catalysts are stable in near-neutral and alkaline conditions but suffer from leaching in acid media. Besides, the pH of the electrolyte represents proton availability. The proton source in the low-pH environment is hydronium (H_3_O^+^), while it is water in the high-pH electrolyte. The local pH can significantly affect the electrochemical ORR via different mechanisms. Yang et al. examined the pH-dependent ORR performance of Ag, Ag-Hg, Pt-Hg catalysts, and the glassy carbon electrode [28]. In the alkaline electrolyte, metal-based catalysts presented lower H_2_O_2_ selectivity but higher 4e^–^ activity than in the acid solution. Glassy carbon exhibited higher activity in alkaline solution but similar selectivity regardless of the electrolyte pH. The author proposed that the kinetic barriers for breaking the O–O bond on metal-based catalysts was higher in acid media, resulting in a more selective H_2_O_2_ generation. As for carbon-based catalysts with weak *OOH interaction, the selectivity was less influenced by the electrolyte pH, but the activity was limited in the acid media.

**Fig. 15:**
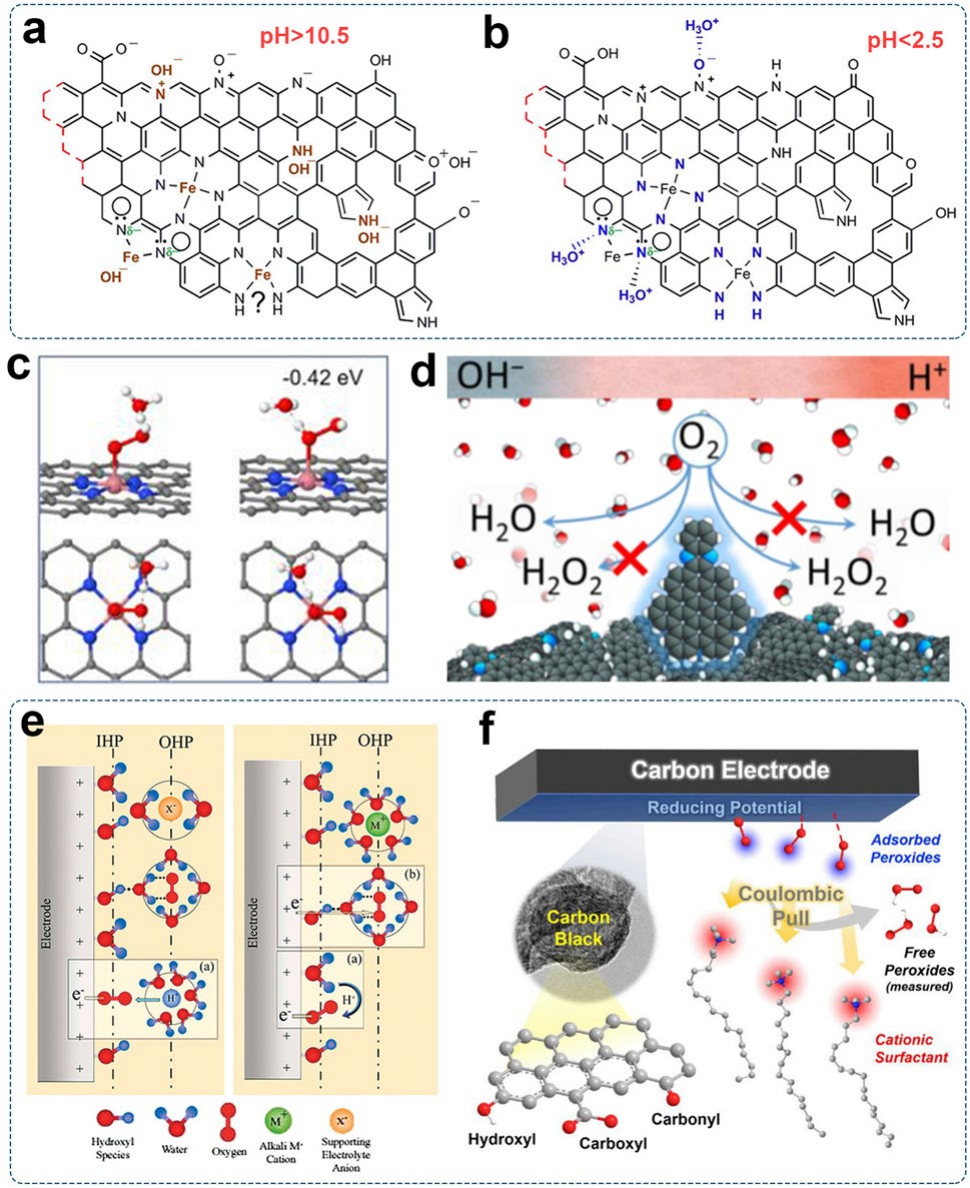
The electrolyte effects: Schematic of the suggested surface chemistry for the Fe−N−C catalyst in **a** highly alkaline (pH > 10.5) and **b** acidic (pH < 2.5) media. Reproduced with permission [211]. Copyright 2018, American Chemical Society. **c** Atomic structures of H_3_O^+^ bonded with different O in *OOH on Co−N−C. Reproduced with permission [212]. Copyright 2021, American Chemical Society. **d** A model for the pH-dependent selectivity of the ORR catalyzed by N-doped graphitic carbon. Reproduced with permission [213]. Copyright 2016, American Chemical Society. **e** Schematic illustration of the double-layer structure during ORR in acidic (left) and alkaline (right) conditions. Insets illustrate the (a) inner- and (b) outer-sphere electron transfer processes. Reproduced with permission [214]. Copyright 2016, American Chemical Society. **f** Schematic illustration of the ORR selectivity modulation by surface-acting cations at the carbon surface. Reproduced with permission [215]. Copyright 2020, Elsevier

The excess of either hydronium ions (H_3_O^+^) in acid media or OH^−^ in alkaline solutions can result in specific modification of the catalyst surface, thereby leading to activity and selectivity changes. Plamen et al. investigated the impact of pH on the chemical state of Fe–N_*x*_ sites toward ORR [211]. They proposed that the adsorbed OH^−^ on the positively charged atoms of Fe–N–C catalyst in high pH electrolyte would serve as inhibitors of oxygen binding, constraining the ORR toward the 2e^–^-pathway (Fig. 15a). When the pH values were below 10.5, protons were available at the catalyst surface, thus neutralizing the adsorbed OH^−^ (Fig. 15b), enabling Fe–N_*x*_ as active centers for the 4e^–^-ORR. A recent computational study proposed that the pH-dependent selectivity of Co−N−C catalysts might be rooted in the proton affinity to the former O in *OOH intermediate [212]. In the acidic condition, the adsorption H_3_O^+^ on the former O in *OOH could increase the H_2_O_2_ selectivity. Oppositely, if the proton preferred the latter O, O−OH breaking would occur, resulting in a low H_2_O_2_ selectivity (Fig. 15c).

Based on theoretical and experimental investigations, Chai et al. proposed a different mechanism from the traditional O_2_ adsorption mechanism for carbon materials in acidic media, in which the first step was hydrogenation of the catalytic site [216]. Since the kinetic barrier for H abstraction was much lower than that for O_2_ adsorption, O_2_ molecules would extract H from carbon materials, generating OOH^−^, which would be neutralized by H^+^ and form H_2_O_2_. It was found that such a mechanism was more thermodynamically favorable than *OOH radical mechanism and more kinetically favorable than O_2_ adsorption mechanism. With the increase of the pH value, the hydrogenation was inhibited, and the O_2_ adsorption mechanism would gradually dominate the ORR, thus leading to the 4e^–^-route.

Noffke et al. proposed a catalytic model based on interfacial solvation and dielectric constant to explain the pH-dependent selectivity of N-doped graphitic carbon for ORR (Fig. 15d) [213]. The hydrophobic environment around catalytic sites would limit access to water molecules and ordered water structures at the electrode, hence limiting the solvation of HO_2_^−^ and in turn, leading to the 4e^–^-pathway in alkaline media. In acid media, the 2e^–^-pathway through H_2_O_2_ elimination would be thermodynamically allowed by the low dielectric constant of the surroundings.

Despite these, the actual electrocatalytic system is rather complex. The local proton concentration and pH may vary during the electrocatalysis process, rendering a dynamic change in the reaction environment. Some studies also proposed that the ORR activity and selectivity might be associated with changes in the electrochemical double layer and electron transfer mechanisms at the interface between the electrode and electrolyte [214, 217]. As shown in Fig. 15e, the electrochemical double layer is constructed from the inner-Helmholtz plane (IHP) and outer-Helmholtz plane (OHP), which are populated by different electrolyte species (solvated O_2_, anions, and cations) under different pH conditions. The inner-sphere electron transfer mechanism is associated with the chemisorption of O_2_ on the catalyst surface, leading to a direct 4e^–^-ORR pathway without desorption of reaction intermediates [217]. The outer-sphere electron transfer (OSET) mechanism involves the noncovalent hydrogen bonding forces between specifically adsorbed hydroxyl species (*OH) and solvated O_2_ (O_2_ (H_2_O)_n_), responsible for the 2e^–^-pathway [217]. Despite the important role of the electrochemical double layer, its contribution to the electrocatalytic process has been less understood than catalysts.

Markovic et al. pointed out that the covalent and non-covalent interactions in the electrolyte system might affect the electrocatalytic properties [218, 219]. The non-covalent interaction between covalently bonded *OH species in IHP and hydrated alkali metal cations (e.g., Li^+^, Na^+^, K^+^) in OHP would form OH_ad_–M^+^(H_2_O)_*x*_ clusters, which would block the active sites for electrocatalytic reactions [218]. The strength of such non-covalent interaction is dependent on the hydration energies of cations (Li^+^  > Na^+^  > K^+^ > Cs^+^), leading to the varied concentration of OH_ad_–M^+^(H_2_O)_*x*_ clusters in electrolytes with different cations. The ORR activity of the Pt catalyst decreases in the sequence of CsOH > KOH > NaOH >> LiOH [218]. Recently, Wang et al. reported a cation-regulated interfacial engineering approach to improve the catalytic performance of 2e^–^-ORR [220]. A small number of Na^+^ cations was added into the acid solution, leading to dramatically improved selectivity and stability of commercial carbon black toward H_2_O_2_ generation. The molecular dynamics simulations suggested that Na^+^ cations could preferentially be attracted to the catalyst-electrolyte interface, generating a “shielding effect” to squeeze out local protons. Thus, the further dissociation of generated H_2_O_2_ (Eq. 5) could be depressed, resulting in better H_2_O_2_ selectivity.

The identity of electrolyte anions (e.g., ClO_4_^−^, SO_4_^2−^, PO_4_^2−^, Cl^−^, Br^−^) is also known to affect the ORR performance of Pt. They would compete with O_2_ for adsorption on the active site, thus altering the binding energy of oxygen intermediates [221]. Nonetheless, it still remains unclear if the anion poisoning can be extended to other catalysts, considering various results observed on M–N–C electrocatalysts [222, 223].

In alkaline media, the anionic HO_2_^−^ that is negatively charged and adsorbed on the catalyst surface would be attracted by the positively charged cations in the bulk electrolyte. The desorption of anionic peroxides could be facilitated via Coulombic attraction induced by a cationic surfactant, Cetyltrimethylammonium bromide (CTAB), hence impeding the further reduction of surface peroxide (Fig. 15f) [215]. The addition of CTAB in the electrolyte significantly improved the kinetics for H_2_O_2_ production while impeding further reduction at the solid–liquid reaction interface, achieving over 95% selectivity across the potential window from −0.1 to 0.7 V (vs. RHE) by using a metal-free CB electrode. Gyenge and Oloman revealed that the cationic surfactant (tricaprylmethylammonium chloride) molecules increased the local pH of the catalyst surface [224]. The nonionic (Triton X-100) and anionic surfactants (sodium dodecyl sulfate) would presumably form surface aggregates and block the access of O_2_ to the catalyst surface, thus rendering the 2e^–^-pathway. These works demonstrate the addition of surfactants for promoting H_2_O_2_ production.

Impurities/contaminants within the electrolyte can be adsorbed and/or deposited on the electrode surface during the electrochemical operation, which may poison the active sites and reduce the activity and selectivity toward the desired reaction route. Contaminates may also come from metal-containing electrocatalysts due to catalyst leaching. The trace amount of metal impurities (e.g., Fe, Mn, Cu) in the electrolyte can catalyze the decomposition of H_2_O_2_, causing production loss [201]. To address the decomposition of H_2_O_2_, some additives, such as diethylenetriaminepentaacetic acid (DTPA), ethylenediaminetetraacetic acid (EDTA), organic phosphonates, and silicates, have been suggested to add into the electrolyte, known as H_2_O_2_ stabilizers [201]. They could deactivate metal impurities and slow down the decomposition reactions.

Overall, electrolyte variations bring about chemical and physical differences in the local reaction environment, which can affect the catalytic properties via multiple mechanisms. The evolution of the catalyst surface chemistry induced by different pH environments, anion/cation compositions, additives, and corresponding impacts on the 2e^–^-ORR performance has not been systemically identified. The fundamental understanding of the electrolyte effect and catalyst-electrolyte interface in the electrocatalysis process still needs further investigation.

## Device Setups and Applications of Electrochemical H_2_O_2_ Production

**Fig. 16:**
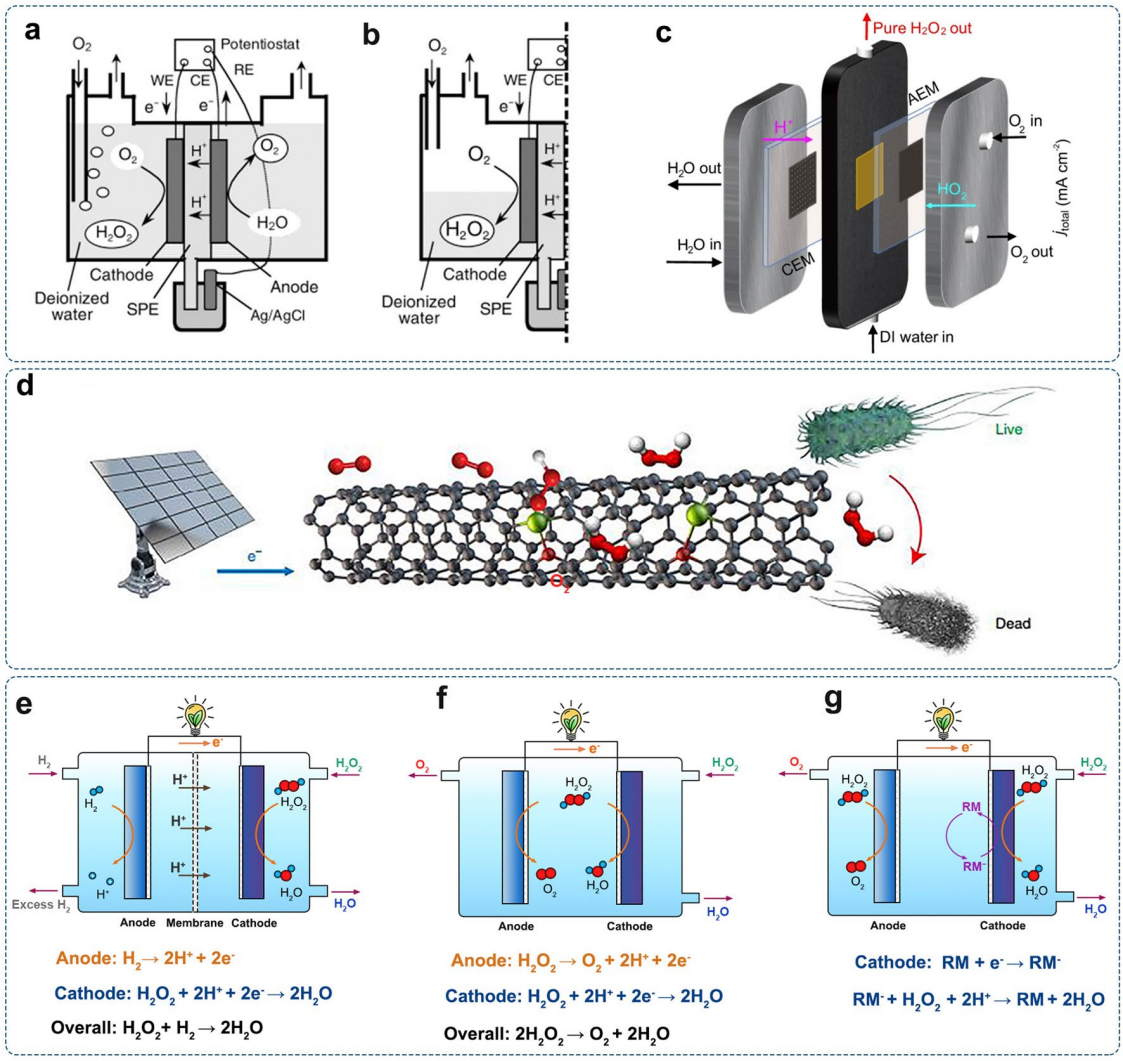
Device setups and applications of electrochemical H_2_O_2_ production. **a–b** Schematic of H-type H_2_O_2_ electrosynthesis cell. Reproduced with permission [3]. Copyright 2008, Wiley–VCH. **c** Schematic of the flow-cell-type H_2_O_2_ generation reactor. Reproduced with permission [116]. Copyright 2021, Springer Nature. **d** Schematic diagram of the electrochemical synthesis of H_2_O_2_ for water disinfection, with green inputs such as sunlight, air, and water. Reproduced with permission [228]. Copyright 2019, Spring Nature. Schematic illustration of H_2_O_2_ fuel cells and corresponding working principles: **e** Two-compartment H_2_O_2_ fuel cell separated by a membrane. **f** One-compartment H_2_O_2_ fuel cell without a membrane. **g** One-compartment H_2_O_2_ fuel cell with a redox mediator

In laboratory research, RRDE setup enables rapid screening of candidate electrocatalysts. However, the maximum current density (only a few mA cm^−2^) and H_2_O_2_ production rate (a few mg h^−2^ cm^−2^) on RRDE are far from the industrial level [225]. Although H-type cells can simulate bulk electrosynthesis of H_2_O_2_ with a current density of tens of mA cm^−2^ (Fig. 16a), the production rate is limited by the low concentration (1 mM) and low diffusion coefficient of aqueous O_2_. To overcome the mass transport limitation, Yamanaka et al. proposed a half-filled cathode chamber in which O_2_ was supplied from the exposed part of the electrode and reacted at the gas-catalyst-electrolyte boundary (Fig. 16b) [3]. In recent years, immense progress has been achieved in developing gas diffusion electrodes (GDE) and flow devices. The flow reactors enable continuous circulation of reactants and products, helping the H_2_O_2_ accumulation to molar concentrations without compromising selectivity (Fig. 16c) [116]. For example, Jaramillo et al. utilized such a reactor for electrochemical H_2_O_2_ production [226]. Scalable electro-production of H_2_O_2_ can be achieved at industrial-level current densities. By tuning the electrolyte flow rate, H_2_O_2_ outflow can be obtained with tunable concentration for different application scenarios [227]. Recent studies have shown the potential applications of electrochemically produced H_2_O_2_ in water treatment, disinfection, and energy storage and conversion.

### Water Treatment and Disinfection

The toxic organic pollutants (e.g., dyes and phenolic compounds) in wastewater pose a considerable risk to human health and the ecosystem [229]. The in-situ generated H_2_O_2_ at the cathode can be combined with certain metal species (e.g., Fe^2+^), ultraviolet radiation, or ozone to form ·OH, which is a powerful oxidizing radical to non-selectively break down most organics and convert them into small molecules or CO_2_. Such an advanced oxidation process is effective for water disinfection. For example, Cui et al. designed an electrochemical reactor for H_2_O_2_ production with oxidized Super P carbon black (O-SP) as a 2e^–^-ORR catalyst. It could generate 10 g L^−1^ H_2_O_2_ solution at the working current of 100 mA and electrolyte (0.1 M Na_2_SO_4_) flow rate of 5 mL h^−1^ [230]. The Fenton filter composed of Cu single atoms incorporated in graphitic carbon nitride (Cu-C_3_N_4_) could activate H_2_O_2_ to generate ·OH. The wastewater treatment system was assembled from the above H_2_O_2_ reactor and Fenton filter. The prototype experiment test showed that the system could treat 270 mL of synthetic wastewater via continuous operation for 100 h, providing a promising strategy for energy-efficient effluent disposal. The residual H_2_O_2_ in the effluent could be controlled below the detection limit (0.05 ppm) by using the Fe_3_O_4_-carbon filter.

In addition, the in-situ generated H_2_O_2_ is a crucial disinfectant against viruses and bacteria (Fig. 16d). The Fe-CNT catalyst demonstrated a rapid disinfection efficiency for *Escherichia coli*, delivering a 43% bacteria inactivation in 5 min and more than 99.9999% in 120 min with no recovery observed [228]. These cases demonstrate the great potential of on-site electrochemical H_2_O_2_ production to develop advanced treatment technologies for future urban, medical, and industrial water systems.

### Energy Storage and Conversion

H_2_O_2_ is a carbon-free energy carrier that can be utilized as both the oxidant and reductant to generate electricity via the fuel cell setup. In a typical H_2_−H_2_O_2_ fuel cell (Fig. 16e), the theoretical open-circuit voltage (OCV) can reach 1.78 V, much higher than these of methanol−O_2_ (1.21 V) and H_2_−O_2_ (1.23 V) systems. Moreover, the cell performance is substantially enhanced with at least 70% higher energy density than that of the H_2_−O_2_ system, together with 5 times higher volumetric energy density than the standard pressurized O_2_ system at 20 MPa [231]. When H_2_O_2_ is used as both oxidant and reductant, the cell can be assembled from one compartment without a membrane (Fig. 16f), thus reducing the ohmic resistance and costs of additional components [4]. The oxidation of H_2_O_2_ to O_2_ at the anode and the reduction of H_2_O_2_ to H_2_O at the cathode can generate a theoretical OCV of 1.09 V [232].

Importantly, H_2_O_2_, as the alternative fuel/oxidant, has significant advantages. Compared with H_2_, H_2_O_2_ can be easily stored at room temperature in a high concentration without additional pressure. As an O-rich reagent, the oxygen-storage capacity of H_2_O_2_ is ∽1600 times higher than in the atmosphere [233]. The storage amount by weight is comparable to the high-pressure oxygen cylinder with 15 MPa, in which 90% of the weight comes from stainless steel cylinders [234]. In contrast to traditional O_2_/air-based fuel cells, the cell system with H_2_O_2_ as an oxidant can also be operated in oxygen-free environments such as outer space and underwater. When H_2_O_2_ is utilized as the reductant or oxidant, anode/cathode materials are employed for catalyzing the H_2_O_2_ oxidation/reduction, respectively. However, it is possible for H_2_O_2_ to be reduced and oxidized on the same surface, resulting in the decomposition of the H_2_O_2_ and generating a mixed potential effect on the electrode [235]. For selectively reducing H_2_O_2_ at the cathode, soluble redox mediator couples (RM/RM^−^) can be introduced. In this case, the cathode chemistry starts with the reduction of the RM to RM^−^. The RM^−^ then participates in the reduction of H_2_O_2_ to H_2_O with the regeneration of RM (Fig. 16g).

Another electrochemical energy conversion device is the metal–H_2_O_2_ cell driven by the electromotive force between metal oxidation and H_2_O_2_ reduction. Xu et al. demonstrated a Zn−H_2_O_2_ cell [234]. The anode reaction in the alkaline electrolyte is the same as the Zn−air batteries (Zn + 4OH^−^  → (Zn(OH)_4_)^2−^  + 2e^−^), while the decomposition of H_2_O_2_ (Eq. 6) at the cathode can provide the oxygen source. Thus, the overall reaction of the Zn–H_2_O_2_ battery can be described as Zn + H_2_O_2_ + 2KOH → K_2_Zn(OH)_4_. The author believed that O_2_ transport in the Zn_2_–HO_2_ battery was faster than that of the Zn−air battery due to the high concentration of O_2_ evolved from the decomposition of H_2_O_2_. As a result, the maximum power density of the optimized Zn − H_2_O_2_ battery is up to 274.1 mW cm^−2^ at 401 mA cm^−2^. Impressively, the assembled Zn−H_2_O_2_ battery could be operated underwater, presenting a significant advantage of this prototype battery. Jun et al. designed a Zn−H_2_O_2_ flow cell in which the V^2+^/V^3+^ and VO_2_^+^/VO^2+^ redox couples worked as the redox mediator at the anode and cathode, respectively [236]. At the anode, the V^2+^ ions were oxidized to V^3+^. The released electrons arrived at the cathode and were accepted by VO_2_^+^, generating VO^2+^. The resultant V^3+^ and VO^2+^ ions then reacted with Zn and H_2_O_2_ in their respective reactors, regenerating V^2+^ and VO_2_^+^ ions. Remarkably, this novel fuel cell displayed an extraordinarily high peak power density of 1,192 mW cm^−2^ at 60 ℃, much higher than state-of-the-art H_2_−O_2_ fuel cells and Zn−air batteries.

It is foreseeable that electrochemical H_2_O_2_ production reactors can be conjugated with these energy conversion systems to provide reactant feeding to generate renewable electricity. The conversion of chemical energy to electric energy can lessen carbon emissions. The reduced costs associated with producing, transporting, and storing H_2_O_2_ also contribute to developing the low-carbon economy.

## Summary and Outlook

The electrochemical 2e^–^-ORR provides a creative and promising way to produce H_2_O_2_. We have reviewed the research progress on the 2e^–^-ORR, including basic mechanisms, experimental and theoretical evaluation methods, catalyst development, and design strategies. Various promising electrocatalysts have been discussed, including noble metals and their alloys, carbon-based materials, transition metals, single-atom, and molecular catalysts. How the developed design strategies have been applied to regulate these catalyst materials’ electronic and geometric structures toward the 2e^–^-ORR has been illustrated based on experimental and theoretical studies. Throughout, controversial issues resulting from measurement setups, catalyst surfaces, and reaction media have been discussed based on the present understanding. Device setups and potential applications of electrochemical production of H_2_O_2_ via 2e^–^-ORR in environment and energy fields have been introduced to minimize the gap between fundamental research and practical implementation.

It is expected that lessons learned from the 2e^–^-ORR to H_2_O_2_ can be applied in other selective electrocatalysis reactions toward value-added chemical products (CO_2_ and N_2_ reduction reaction) in terms of reducing the competing side reactions and enhancing the Faradaic efficiency. Despite enormous progress, this promising electrochemical technology is still in its infancy and has a long path toward its applications on the practical level. The following aspects and research directions demand to be focused on:

### Searching for Optimal 2e^–^-ORR Electrocatalysts

Due to the base-catalyzed degradation, H_2_O_2_ is less stable in alkaline conditions, which may limit its applications. Therefore, future efforts should focus on exploring electrocatalysts that can effectively drive the 2e^–^-ORR in acidic or neutral conditions. Meanwhile, the previous 4e^–^-ORR studies have investigated a wide range of catalyst materials. This means that ORR catalysts that are less efficient for fuel cells could be re-examined and potentially used for the electrosynthesis of H_2_O_2_.

Beyond the trial-and-error approach, the well-established artificial neural network combined with the data obtained from experimental measurements and first-principal calculations might help identify the most desirable structural features of catalyst materials for targeted reactions. In this way, the computation-experiment cycle time will be significantly reduced, thus accelerating the discovery of high-performance electrocatalysts. However, the accuracy and performance of data-driven algorithm models depend heavily on the quality and scale of the database since unbalanced data would lead to underfitting problems and biased predictions.

Electrocatalysts fabricated from earth-abundant raw materials are highly desirable for practical-scale applications. Meanwhile, sustainable fabrications, such as green chemistry approaches and substitution of hazardous materials with more environmentally friendly alternatives, are essential for reducing the generation of toxic and harmful chemicals and alleviating the environmental impact of catalyst production. In this way, electro-synthesis could pave an efficient and green way for H_2_O_2_ production.

### Catalytic Stability

Although the activity and selectivity issues are urgent to be solved, the long-term stability of catalysts is also critical for maintaining robust and sustainable H_2_O_2_ production. The assessment of catalytic stability in most studies normally lasts for several or several tens of hours, which is too short to convincingly manifest long-term durability. Moreover, as produced H_2_O_2_ needs to be accumulated in useful concentrations over the continuous operation, catalysts materials should maintain stability under the harsh oxidizing conditions due to the powerful oxidizing ability of the formed product. Thus, prolonged durability tests (e.g., > 100 h) in a high-concentration H_2_O_2_ environment (e.g.,  > 3 wt%) at high current density (e.g., > 100 mA cm^−2^) need to be carried out along with the post characterizations to study the durability of catalysts under more realistic operating conditions. In order to reduce the capital cost of replacing electrodes and reactor downtime, the dissolution/detachment issues, structure and composition stability, and electrochemical process-induced surface changes of electrocatalysts should also be considered more seriously.

### Further Understanding of the ORR Mechanism

To date, the fundamental understanding of the ORR mechanisms catalyzed by electrocatalysts toward H_2_O_2_ is still insufficient. Based on the examples discussed herein, many works investigate the 2e^–^-ORR process in alkaline or acidic electrolytes, while the ORR in neutral media is far less studied. H_2_O_2_ produced in neutral aqueous can be flexibly tailored for specific applications with various pH environments. However, the limited availability of both H^+^ and OH^−^ in the neutral media may be the bottleneck. The cost-effective 2e^–^-ORR electrocatalysts and corresponding mechanisms under the neutral condition, an important frontier ahead, are still being explored.

Apart from the electronic and geometric structures of the catalysts, the catalytic performance of 2e^–^-ORR is also affected by many other factors, such as electrolytes (e.g., pH, buffer cations/anions, proton sources, and concentrations), the interfacial structure between electrolyte and catalyst, applied potentials, impurities, and other external influences. However, many of these factors and their potential impacts lack comprehensive studies in both theory and experiments. Importantly, most results obtained from theoretical approaches typically focused on a few key properties of the catalyst surface and simplified the calculation of adsorption energies under the vacuum condition. This is possibly not sufficient for elucidating the complex electrochemical reactions under operating conditions. New modeling approaches regarding the electrode–electrolyte interface and dynamic behavior of the catalyst during different operating stages are essential goals for future efforts but remain a great challenge. The same holds for the development of more advanced experimental methods that can fabricate ideal model catalysts to study structure–property relationships with fewer interferences.

### Advanced Characterization Techniques in the Electrochemical System

The comprehensive and multifaceted experimental evidence is helpful in uncovering the catalytic behavior of catalysts and gaining deeper insights into the reaction mechanism. Multiple operando characterization techniques are suggested to study the dynamics phenomena occurring at the electrode/electrolyte interface under operating conditions. With the elaborately designed reactors and instrumentation setup, the in-situ synchrotron X-ray techniques (e.g., XPS and XAS) have been commonly employed to monitor the chemical states of the catalyst surface in liquid environments under operating conditions. They serve as the most comprehensive tools available for experimentally identifying the active sites and investigating chemical changes of catalysts toward reaction intermediates. In-situ microscopic techniques are powerful tools that enable researchers to visualize structural changes of solid catalysts under liquid environments, providing valuable insight into catalyst structure–reactivity relationships under realistic electrochemical conditions. In particular, the in-situ TEM has been used to monitor the evolution of material surface morphology and crystallinity with nanoscale or even atomic spatial resolution in real-time.

Similarly, the adsorbed species on the catalyst surface during ORR demand operando observations so that the catalytic reaction mechanisms can be understood more comprehensively. Surface-enhanced Raman spectroscopy (SERS) is a useful and practical tool that can probe catalyst-adsorbate interactions in electrochemical systems with weak Raman scatterers. Numerous catalysts have been studied with in-situ SERS to identify adsorbed intermediates and study the chemical changes of catalysts, therefore further understanding electrochemical reaction mechanisms. The continuous innovation of in-situ characterization and analytical techniques of electrochemical systems will provide more informative results and make an exciting breakthrough.

### Design of Reactor Systems

The developed strategies to engineer catalyst materials are just the first step in opening up opportunities for sustainable electrosynthesis of H_2_O_2_. For industrial-scale applications, other factors affecting the H_2_O_2_ production rate should be considered, including electrode materials (substrates, binders, and current collectors), electrolytes, ion exchange membrane, reactor configurations, and operation conditions (e.g., temperature, flow rate, the applied potential/current, and the O_2_ partial pressure). Electrochemical reactor systems should consider the energy costs for electrocatalytic operation and circulating the gas and electrolyte to reduce manufacturing costs. The reactor components should have good resistance against the corrosion from the electrolyte and H_2_O_2_. Meanwhile, the reactor design should enable the facile separation and stabilization of H_2_O_2_. However, the development of electrochemical reactor system design is relatively slow compared to the catalyst design. Although flow cells have gained significant interest in electrochemical applications, there are also some technical issues, such as the high cost of electrode materials and manufacturing processes, the destruction of triple phases, uneven conversion efficiency, and the peeling-off of active species. Innovative device designs are needed to make electrochemical H_2_O_2_ production more cost-effective.

Moreover, the integrated anodic half-reactions (e.g., hydrogen oxidation reaction, oxygen evolution reaction, and organic oxidation reactions) also play a crucial part in the overall electrocatalysis conversion process. Its progress will maximize the energy efficiency for converting earth-abundant raw materials toward high-value-added chemical products. Undoubtedly, joint efforts are demanded in various fields to optimize the electrochemical system.
